# Impact of N-heterocyclic amine modulators on the structure and thermal conversion of a zeolitic imidazole framework†

**DOI:** 10.1039/d5ta04831a

**Published:** 2025-07-24

**Authors:** Javier Castells-Gil‡, Jinjie Zhu‡, Ioanna Itskou, Emma H. Wolpert, Robert D. Hunter, Jeremiah P. Tidey, Angus Pedersen, Elisa Solvay, Helen Tyrrell, Camille Petit, Jesús Barrio

**Affiliations:** a School of Chemistry, University of Birmingham Birmingham B152TT UK j.castells-gil@bham.ac.uk; b Department of Chemical Engineering, Imperial College London London SW27 2AZ UK j.barrio-hermida@imperial.ac.uk; c Department of Chemistry, University College London London UK; d Department of Physics, University of Warwick Coventry CV4 7AL UK; e Bundesanstalt für Materialforschung und -Prüfung (BAM) 12203 Berlin Germany

## Abstract

The zeolitic imidazole framework-8 (ZIF-8) is a crystalline porous material that has been widely employed as template to fabricate porous nitrogen-doped carbons with high microporosity *via* thermal treatment at high temperatures. The properties of the carbon scaffold are influenced by the pore structure and chemical composition of the parent ZIF. However, the narrow pore size distribution and microporous nature from ZIF-8 often results in low mesopore volume, which is crucial for applications such as energy storage and conversion. Here we show that insertion of N-heterocyclic amines can disrupt the structure of ZIF-8 and dramatically impact the chemical composition and pore structure of the nitrogen-doped carbon frameworks obtained after high-temperature pyrolysis. Melamine and 2,4,6-triaminopyrimidine were chosen to modify the ZIF-8 structure owing to their capability to both coordinate metal ions and establish supramolecular interactions. Employing a wide variety of physical characterization techniques we observed that melamine results in the formation of a mixed-phase material comprising ZIF-8, Zn(Ac)_6_(Mel)_2_ and crystallized melamine, while 2,4,6-triaminopyrimidine induces the formation of defects, altering the pore structure. Furthermore, the absence of heterocyclic amine in the ZIF-8 synthesis leads to a new crystalline phase, unreported to date. The thermal conversion of the modified ZIFs at 1000 °C leads to nitrogen-doped carbons bearing Zn moieties with increased surface area, mesopore volume and varying degree of defects compared to ZIF-8 derived carbon. This work therefore highlights both the versatility of heterocyclic amines to modify the structure of framework materials as well as their role in tuning pore structure in nitrogen-doped carbons, paving the way to targeted design of high-performance electrodes for energy storage and conversion.

## Abstract 2

Heterocyclic amines modify the structure of ZIF-8, yielding a defective ZIF, a mixed-phase material, and a new layered ZIF phase. These changes enhance porosity and defect levels in the resulting nitrogen doped carbons.

## Graphical abstract

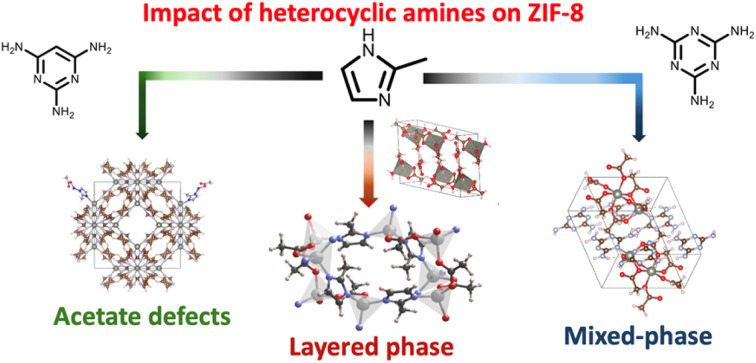

## Introduction

Zeolitic Imidazolate Framework – 8 (ZIF-8) is a metal–organic framework (MOF) with a zeolite-like topology which is formed by Zn^2+^ ions coordinated to methyl imidazole (MIm) ligands,^1^ albeit analogues with Co^2+^, Fe^2+^ or Mg^2+^ metal centres can also be found.^2–4^ Their intrinsically high microporosity and thermal and chemical stability has led to their wide implementation in gas sorption and separation,^5,6^ catalysis,^7^ and as a template to prepare microporous nitrogen-doped carbon materials *via* high-temperature pyrolysis, owing to their chemical composition.^8,9^ To date, plenty of modifications in the synthesis protocol of ZIF-8 have been explored in terms of Zn^2+^ salt, solvent, temperature or reaction time, which lead to variations in particle size and porosity,^10,11^ and whose impacts have been recently disclosed *via* text mining and meta-analysis.^12^ Additionally, the versatility of the ZIF family allows the utilization of alternative organic linkers and even the formation of multivariate MOFs.^13^ Depending on the nature and coordinating ability of the building blocks employed in the synthesis, the organic ligands can either take part in the framework or act as modulator to create defects.^14^ For instance, Falcaro and co-workers employed 20 amino acids to facilitate the crystallization of ZIF-8 and correlated the morphology and size of the resulting crystals to the employed amino acid.^15^ An and co-workers employed amine modulators such as butylamine or tributylamine to tune the pore size distribution and improve the C_3_H_6_/C_3_H_8_ separation performance. The utilization of Zn(ii) acetate allowed the suppression of nuclei formation, allowing the amine modulators to coordinate with Zn^2+^ ions without altering the ZIF-8 unit cell.^16^ Similar results were obtained recently by Tivanski and co-workers who employed Et_3_N as a supramolecular modifier in the ZIF-8 synthesis. Using this modifier led to change of the elastic modulus owing to a lower relative number of hydrogen bonds and methyl imidazole ligands compared to the parent ZIF-8.^17^

The partial replacement of MIm ligands by building blocks capable of both coordinating Zn^2+^ ions and interact *via* supramolecular interactions (such as hydrogen bonding) can yield new porous frameworks as exemplified recently by Baslyman *et al.*^18^ The coordination of Zn^2+^ ions with MIm and adenine ligands resulted in the formation of a hydrogen bonded framework, where zinc adeninate macrocylces self-assemble into a 3D framework *via* H-bonding.^18^

One of the potential benefits of modifying the ZIF-8 crystal structure with coordinating organic building blocks entails the impact of the pore structure of the resulting carbon material upon thermal conversion. ZIF-8 can be converted into highly microporous nitrogen-doped carbon by pyrolysis at >700 °C and therefore has been widely explored as a precursor in the field of electrocatalysis with carbon materials.^19–22^ The narrow pore size imprinted by the microporous nature of ZIF-8 brings limitations in terms of active site accessibility in electrocatalysis,^23,24^ and therefore synthetic approaches that provide certain mesopore volume in ZIFs are sought after. So far, this has been achieved *via* top-down approaches such as clip-off chemistry or acid etching,^25–27^ although recently Liu *et al.* reported the synthesis of mesoporous ZIF-8 crystals *via* self-assembly with a block copolymer.^28^

Amongst the organic linkers that one could envision to coordinate with Zn^2+^ ions while providing supramolecular functionality, N-heterocyclic amines emerge as suitable candidates. Melamine (2,4,6-triamino-*s*-triazine, Mel), for instance, is a heterocyclic compound which can interact *via* hydrogen bonding as well as π–π stacking and coordination bonds owing to its three amine groups and three sp^2^ nitrogen atoms, consequently providing a wide range of possibilities to synthesize supramolecular and metal–organic materials.^29–32^ In fact, Mel has been shown to form organic–inorganic hybrid materials *via* coordination to Zn^2+^, highlighting its potential to render supramolecular functionality in ZIF frameworks.^33^ Furthermore, Mel and its derived supramolecular and hybrid materials have been widely employed as reactants to construct carbon–nitrogen based materials,^34–36^ as well as dopants in graphene-based catalysts to induce nitrogen functionalities.^37–40^ To our knowledge, the utilization of Mel and similar N-heterocyclic amines (such as 2,4,6-triaminopyrimidine, Tap) to modulate the crystalline phases of ZIF-8 has not been reported to date. Therefore, in this work we explore the impact on the ZIF-8 synthesis and crystal structure of melamine and 2,4,6-triaminopyrimidine and study the chemical and physical properties of the obtained structures as well as those of the carbon materials obtained *via* pyrolysis at high temperature. We observed that the utilization of 2,4,6-triaminopyrimidine induces structural defects on ZIF-8, which decrease the pore size and increase the BET surface area. On the other hand, melamine coordinates to Zn^2+^ ions, driving the formation of a mixed phase material. Such properties dramatically influence the pore structure of the nitrogen-doped carbons obtained after pyrolysis at 1000 °C, inducing significant mesopore volume.

## Experimental section

### Synthetic procedures

#### MOF synthesis

ZIF-8 was synthesized as previously reported.^41^ Briefly, 0.297 g (1 mmol) of zinc nitrate hexahydrate were dissolved in 11.3 mL of methanol, followed by the addition of 0.656 g (8 mmol) of 2-methylimidazole in 11.3 mL of methanol at room temperature. The mixture was stirred for 24 h at room temperature, centrifuged at 8000 rpm for 10 min and the resulting powder washed with methanol twice. The resulting ZIF-8 powder was dried overnight at 80 °C in an oven. Melamine and Tap-modified ZIFs were synthesized by mixing 10 mmol of either melamine or Tap (1.26 and 1.35 g, respectively), 820 mg of methylimidazole (10 mmol) and 2.19 g of Zn acetate dihydrate (10 mmol) with 200 mL of IPA in a round-bottom flask at 70 °C for 24 h under vigorous stirring. After 24 h, the stirring was stopped, and the solvent removed once the powder had settled at the bottom of the flask. Then 200 mL of hot IPA were added, and the mixture stirred for 2 h. The material was then collected in 50 mL falcon tubes, centrifuged at 8000 rpm for 10 min, the supernatant discarded, and the solid powder dried overnight at 80 °C in an oven.

#### MOF thermal conversion

To convert the prepared MOFs into nitrogen-doped carbon materials, 200 mg of either ZIF-8, Mel-ZIF or Tap-ZIF were placed in a ceramic crucible, and these placed in the middle of a tubular furnace. The crucibles were then heated to 1000 °C at 5 °C min^−1^ in N_2_ atmosphere with a N_2_ flow of 300 mL min^−1^, with a 1 h hold, before cooling naturally. The obtained materials were labelled as ZIF-81000, Mel-ZIF1000 and Tap-ZIF1000.

### Characterization

Fourier Transformed Infrared spectroscopy (FT-IR) was collected in attenuated total reflectance mode with an Agilent Cary 630 FRIF spectrometer after collecting 32 background and sample scans. Powder XRD patterns (with a 0.016° scan step size) were obtained using a PANalytical X’PERT PRO powder X-ray diffractometer equipped with a Cu Kα1/Kα2 source (*λ* = 1.5406 Å), operating at 40 kV and 40 mA, over a 2*θ* range of 5° to 60. Powder XRD patterns with Mo Kα_1_ (*λ* = 0.70926 Å) were collected on a STOE Stadi-P diffractometer equipped with a Ge monochromator, operating at 50 kV and 20 mA. Scans were collected between 2 and 50° (2theta) in a Debye–Scherrer geometry using 0.5 mm borosilicate glass capillaries. ^13^C Solid state NMR was performed in a Bruker Avance III HD 600 MHz spectrometer spinning at 15 000 Hz with a 3.2 mm rotor. Liquid-state NMR spectra were obtained with a Jeol (1H, 400 MHz) spectrometer. 10 mg of ZIF-based material was dispersed in 0.8 mL of D-DMSO and then a drop of 35% DCl in D2O was added to dissolve the material.^42^ An ION-TOF V (IONTOF GmbH, Germany) was used for time-of-flight secondary ion mass spectrometry (ToF-SIMS) measurements, which employed a 100 μm × 100 μm field of view using a Bi^3+^ primary ion beam with 25 keV and 0.34 pA beam current and 256 × 256 pixels in high current bunched mode. Measurements were recorded for 25 min and rastered in sawtooth mode, with 100 μs cycle time (870 amu), employing a 5 μm spot size. The flood gun was on during measurements. Positive spectra were calibrated to: C^+^, CH^+^, C_3_H_5_^+^, Zn^+^, C_5_H_9_^+^, C_7_H_13_^+^, with deviation <70 ppm for all calibrations peaks in samples except Zn^+^ which was 110–130 pm. Data analysis was performed using SurfaceLab 7 software, and normalization of peak intensity counts was computed based on the total ion count across the measured spectrum. All identified peaks show mass resolution <4000 mass units. N_2_ sorption measurements were carried out at 77 K in the pressure range of 10^−5^ to 0.99 with a Micromeritics 3Flex Sorption Analyzer and nitrogen N_6.0_ (99.9999% N_6.0_ Grade N_2,_ BOC). Prior to the N_2_ sorption (77 K) measurements, the samples were *ex situ* degassed for 16 h at 473 K using a Micromeritics SmartVacPrep, and *in situ* degassed at the 3Flex Sorption Analyzer for 16 h at 573 K. The BET area was obtained with the BET surface identification software analysing the adsorption isotherm in the pressure range of 0.995 as determined by the Rouquerol method.^43,44^ The pore size distribution was determined from the adsorption isotherm using the heterogeneous surface carbon 2D non-local density functional theory (NLDFT) method. This approach accounts for the energetic heterogeneity and geometrical corrugation of the carbon surface, thereby providing a more realistic model and avoiding common artifacts. The pore size distribution, ranging from 0.3 to 50 nm, was generated using SAIEUS software (version 3.06). X-ray photoelectron spectroscopy (XPS) was performed in a Thermo Fisher K-Alpha system. The spectra were analysed with the Avantage software, and all the spectra calibrated relative to the C 1s peak at 284.8 eV. Raman spectra were collected on a Renishaw inVia micro-Raman (500–3200 cm^−1^) using a green laser with a wavelength of 532 nm at 10% laser power. Statistical Raman data were obtained from measurements at 5 points per sample. All Raman spectra were normalised by maximum intensity, baseline-subtracted and cosmic rays removed. Spectra were deconvoluted using a four-peak model with Voigt line shapes to extract *I*_D_/*I*_G_ values. The additional D_3_ and D_4_ bands used in the deconvolution procedure have been previously ascribed to amorphous carbon and polyene structures respectively.^45^ Thermogravimetric analysis was carried out with a PerkinElmer TGA8000 with either nitrogen or air environment (with a flowrate of 40 STP mL min^−1^) in a temperature range of 30–900 °C and heating rate of 5 °C min^−1^. Inductively Coupled Plasma Mass Spectrometry (ICP-MS) was performed using an Agilent 7900 spectrometer (Agilent Technologies). For ICP-MS analysis, approximately 5–8 mg of material were first digested in aqua regia (25 v/v% HNO_3_,65%, Certified AR, Eur.Ph., for analysis, Fisher Chemical, Fisher Scientific) and (75 v/v% HCl (37%, Certified AR, Eur.Ph., for analysis, Fisher Chemical, Fisher Scientific)) using a MARS 6 microwave at 1500 W for 15 minutes at 215 °C. The resulting solutions were diluted 300–500 times and measured against calibration standards containing Zn concentrations of 0, 2, 50, 100, 200, and 500 ppb. CO_2_ adsorption isotherms were measured sequentially at 290 K, 299 K and 308 K and up to 1 bar, using a Micromeritics 3Flex Sorption Analyzer. Prior to the CO_2_ measurements, the samples were degassed *ex situ* using a Micromeritics VacPrep overnight at 393 K and at 2 × 10^−5^ bar, and then *in situ* using the 3Flex Sorption Analyzer at 393 K for 4 h down to 7 × 10^−5^ bar. N_2_ adsorption isotherms were measured at 299 K up to 1 bar, right after finishing with the CO_2_ measurements. The samples were *in situ* degassed at 393 K for 4 h down to 7 × 10^−5^ bar. CO_2_ gas (research grade, 99.999%, BOC) and N_2_ gas (99.9999%, BOC) were used for these analyses. The isotherms were fitted using single site Langmuir (SSL) model:1
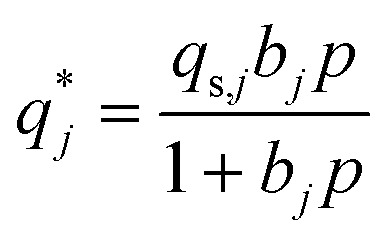
2
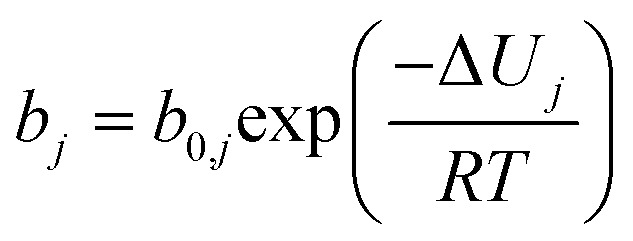
where 
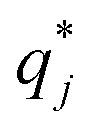
 (eqn (1)) is the adsorbed amount of gas *j* at pressure *p* and temperature *T*, *b* is an adsorption coefficient (eqn (2)), *b*_0_ and Δ*U* are constants, *R* is the universal gas constant, and *q*_s_ is the saturation capacity. The SSL fittings were carried out with MATLAB R2022a (The Mathworks Inc.) using the in-house software package isothermFittingTool.^46^

Afterwards, the isosteric heat of adsorption (Δ*H*_ads_) for CO_2_ was calculated by applying the Clausius–Clapeyron equation using the above SSL parameters,^47,48^ based on the eqn (3):^49^

3 −Δ*H*_ads_ = −Δ*U*_*j*_

### Single crystal 3D electron diffraction

For the 3D ED experiment, the samples were dispersed dry onto copper-supported holey amorphous carbon TEM grids, either dry with prior light grinding (Mel-ZIF) or sonicated in iso-propyl alcohol and drop cast (ZIF-IPA). There were flash frozen in liquid nitrogen and loaded *via* a high-tilt Gatan ELSA cryogenic specimen holder into a Rigaku XtaLAB Synergy-ED electron diffractometer, operated at 200 kV and equipped with a Rigaku HyPix-ED hybrid pixel array area detector. The temperature in the sample airlock was held at 210 K to allow for ice to evaporate, prior to cooling and insertion into the column for collection. Data were collected at 100(5) K and 125(5) K for crystallites of Mel-ZIFs and ZIF-IPAs, respectively, as single-rotation scans collecting 0.25° frames using *CrysAlisPRO* system (CCD 1.171.44.94a 64-bit (release 17-02-2025))^1^ using continuous rotation electron diffraction with a selected area aperture of either 1 or 2 μm apparent diameter. Precise experimental details are provided in Table S3.†

All single crystal component datasets were individually indexed and integrated, with all bar ZIF_IPA_B108 seeing the further merging of multiple datasets and subsequently scaled using *CrysAlisPRO* (version 1.171.44.112a);^50^ no absorption corrections were applied. The structures were solved using ShelXT^51^ and refined using Olex2.refine in the kinematic approximation, applying extinction corrections to broadly account for multiple scattering alongside judicious rejection of outlying reflections, as implemented in Olex2 (version 1.5-ac7-014, compiled 2025.02.27 svn.r6f4c0eaf for Rigaku Oxford Diffraction, GUI svn.r7171)^52,53^ using published scattering factors.^54^

Hydrogens were placed geometrically at neutron average distances and their subsequent refinement precisely described in the CIFs. Non-hydrogen atoms were refined anisotropically, and global rigid bond restraints was employed only in the case Zn(Ac)_6_(Mel)_2_ to improve the physical sense of those displacement parameters. Experimental and refinement information are contained within the deposited CIF along with structure factors and embedded .RES files; structure CIFs are deposited in the CSD with Deposition Numbers 2456572–2456575.

### Rietveld refinements

Rietveld refinements were carried out using the software Topas Academic v7 (https://www.topas-academic.net/).^55^ For the Rietveld refinements, the structural models obtained from ED were used, for which lattice parameters, scale factor, strain and crystallite size were modelled. In the case of the melamine phase, melamine molecules were modelled as rigid bodies. The bond distances were set as 1.38 Å (aromatic C–C/C–N bonds), 1.45 Å (C–N bonds) and all H atoms were placed in ideal position with bond distances of 1.08 Å, which were fixed throughout the refinement. The background was fitted with a 18-coefficient Chebyshev polynomial. The instrumental parameters were obtained from the measurement of a LaB_6_ standard, whose peak-shapes were modelled with a Thompson–Cox–Hasting pseudo-Voigt profile function. Structural visualisation was carried out using the VESTA software.^56^

### Computational details

To investigate the effect of acetate defects on the pore size distribution, we generated ZIF-8 structures with varying defect concentrations (0 defects, 1 defect (4%), 6 defects (25%), 12 defects (50%), 24 defects (100%)). For each defect concentration, we started with the experimentally reported structure of ZIF-8 (ref. 5) and replaced MIm linkers with acetate before optimising the structure and calculating the pore size distribution. The defects were evenly distributed throughout the framework to maintain a near constant ratio of Zn–N bonds per metal centre for each defect concentration. Each structure was optimised using density functional theory (DFT) in the mixed Gaussian and plane wave code CP2K/QUICKSTEP.^57^ We employed the PBE functional with Grimme D3 dispersion corrections,^58,59^ GTH-type pseudopotentials,^60^ and the TZVP-MOLOPT basis set for all atoms except zinc,^61^ for which DZVP-MOLOPT-SR-GTH basis set was used to reduce computational cost.^61^ The optimisations were performed in two stages, first the atomic positions were relaxed with fixed cell parameters and then the cell parameters were optimised. For all calculations, the plane wave cutoff was set to 1350 Ry with a relative cutoff of 70 Ry.

## Results and discussion

**Scheme 1:**
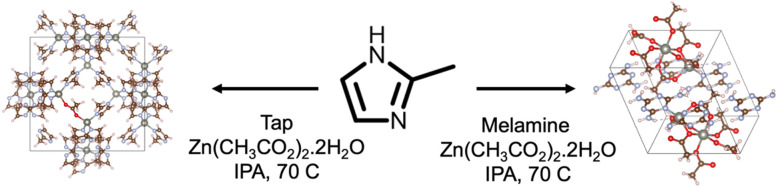
Schematic representation of the Tap-ZIF and Mel-ZIF synthesis.

**Fig. 1:**
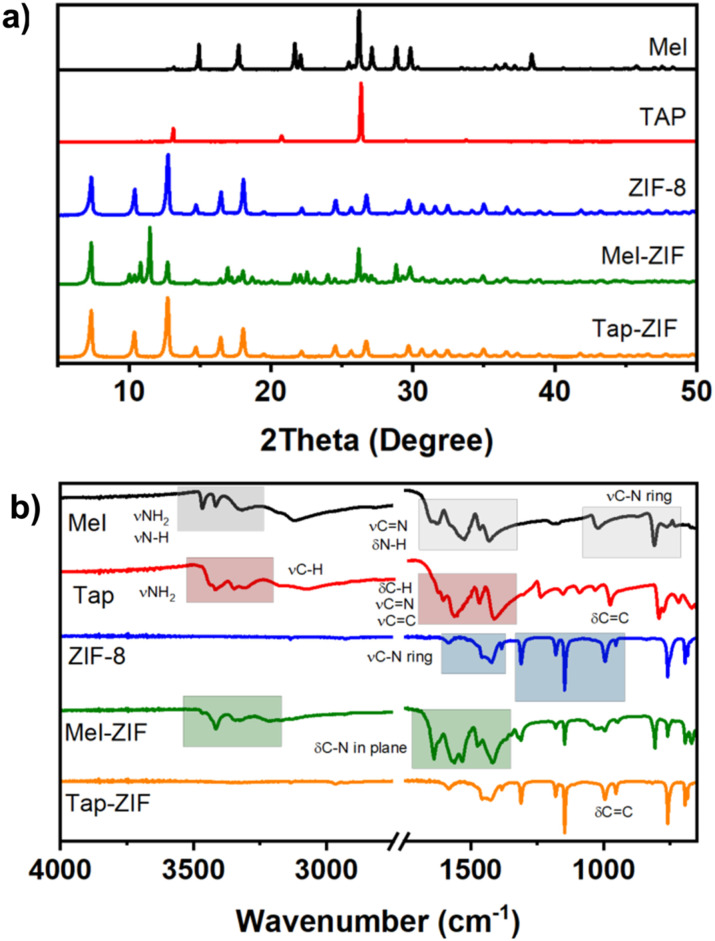
XRD patterns (a) and FT-IR spectra (b) of the prepared ZIF-derived materials and their comparison with melamine and 2,4,6-triaminopyrimidine.

Mel and Tap were chosen as modulators in the synthesis of ZIF-8 owing to their rich supramolecular chemistry as well as their potential to prepare nitrogen-doped carbon materials upon thermal treatment.^24,62^ The synthesis was carried out in IPA at 70 °C using Zn^2+^ acetate as the Zn source instead of Zn^2+^ nitrate to suppress the early nucleation of ZIF-8 crystals,^11^ in a molar ratio of 1 : 1 : 1 Mel/Tap : MIm : Zn (Scheme 1). Powder XRD patterns confirm the successful formation of a ZIF-8 structure and the formation of a new phase in Mel-ZIF (Fig. 1a). In the case of Tap-ZIF, however, no change can be observed between its diffraction pattern and that of ZIF-8. FT-IR further confirms the inclusion of melamine within the structure of ZIF-8 and the absence of Tap vibrations in Tap-ZIF (Fig. 1b). Mel signals can be seen at 3414 cm^−1^ corresponding to the stretching vibrations of N–H groups, as well as at 1600–1400 and 804 cm^−1^ arising from the triazine stretching and breathing vibrational mode. Additionally, the disappearance of one of the N–H vibrations within Mel could point to the formation of intermolecular hydrogen bonding similar to the case of self-assembled zinc adeninate macrocycles.^18,63^

**Fig. 2:**
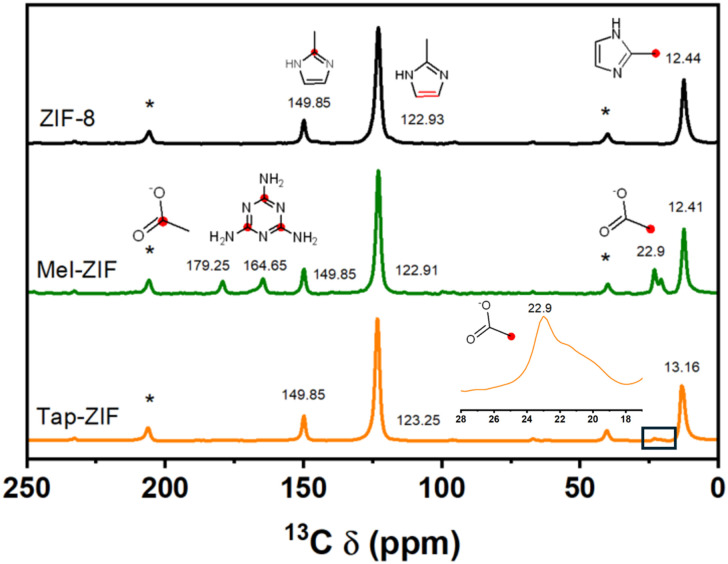
^13^C Solid state NMR spectra of ZIF-8, Mel-ZIF and Tap-ZIF (* denotes the spinning bands and inset shows magnification in the chemical shift range of acetate).

^13^C NMR further supports the inclusion of Mel as observed in the signal located at 164 ppm (Fig. 2). Interestingly, signals corresponding to acetate ligands can also be observed at 22.9 and 179.25 ppm, suggesting that besides nitrogen, Zn^2+^ is also coordinated to oxygen.

**Fig. 3:**
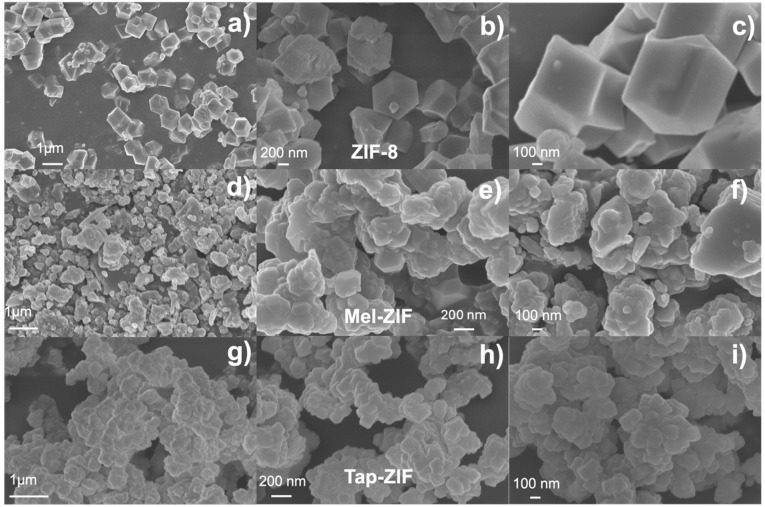
SEM images of ZIF-8 (a–c), Mel-ZIF (d–f) and Tap-ZIF (g–i).

**Fig. 4:**
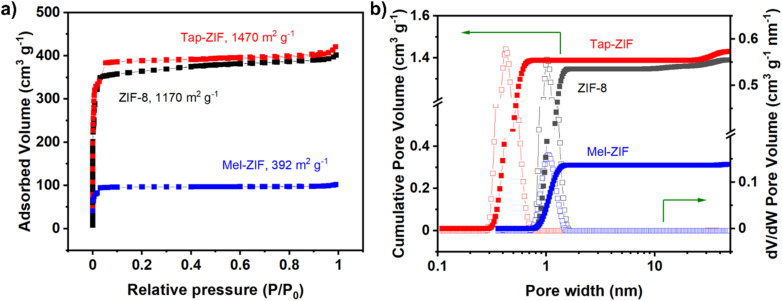
N_2_ sorption (77 K) characterization of ZIF materials. (a) Adsorbed volume and (b) pore size distribution.

As expected, Tap-ZIF displays an identical spectrum to that of ZIF-8 displaying the bands corresponding to the methyl group, sp^2^ carbon and tertiary carbon atoms at 13.16, 123.25 and 149.85 ppm, respectively.^42^ However, the signal corresponding to the –CH_3_ group of acetate can also be observed, albeit with a much lower intensity, suggesting the presence of acetate defects replacing MIm linkers. The morphology of the ZIF-derived materials was analysed by SEM, where substantial differences can be observed in terms of particle size and morphology (Fig. 3). While reference ZIF-8 displays the typical rhombic dodecahedral morphology of around 1 μm size, both Mel-ZIF and Tap-ZIF display irregular shapes with a much smaller particle size. The impact of the distinct morphology on the pore structure of the prepared materials was studied *via* N_2_ sorption experiments (Fig. 4). The N_2_ sorption and pore size distribution of ZIF-8 and Tap-ZIF both exhibit typical Type I behaviour characteristic of microporous materials, with a sharp uptake at low relative pressures. However, Tap-ZIF displays higher BET area (1470 *vs.* 1170 m^2^ g^−1^ for ZIF-8, Fig. S1a†) and a shift in pore size distribution toward smaller pores (∼0.5 nm *vs.* ∼1.0 nm for ZIF-8, Fig. 1b). This behaviour supports the presence of structural missing linker defects in Tap-ZIF and the incorporation of acetate anions (as shown *via* NMR), rather than Tap taking part in the coordination sphere of Zn^2+^ centres. These defects likely generate additional surface area and partially constrict the pore apertures, leading to smaller pore size.^64^

To test this hypothesis, we introduced acetate defects into the ZIF-8 framework and optimised the resulting structures using density functional theory (DFT) calculations with the PBE functional^58^ and Grimme-D3 dispersion correction (see Experimental section for more details).^59^ We then calculated the pore size distribution using Zeo++^65^ across varying levels of defect incorporation (Fig. S1†) from pristine ZIF-8 (0 defects) to 100% defects (*i.e.* complete replacement of MIm ligands with acetate) including 1 defect, 25% and 50%. We note that from the FT-IR spectra (Fig. 1b) it is unlikely that there is a defect concentration is greater than 25%, and higher substitution levels are explored purely for illustrative effects on the trend. The pore size distributions were calculated using the high accuracy flag with 50 000 MC samples and a probe diameter of 1.2 Å.

For ZIF-8, the calculated pore size distribution shows a narrower range and a larger pore width (Fig. S2a,† 1.15 nm) than experiment (approximately 1.0 nm). This discrepancy likely arises from the inherent flexibility of the ZIF-8 framework, which is not captured computationally as Zeo++ calculations are performed on static structures and therefore do not capture framework flexibility. As the defect concentration increases, the pore width decreases, reaching approximately 1.0 nm when all MIm linkers are replaced by acetate. While the magnitude of the decrease in pore size seen computationally is smaller than the experimental change from ZIF-8 to Tap-ZIF, the same general trend is seen, supporting the view that the experimentally observed decrease in pore size arises from a higher defect density. Furthermore, the simulated XRD patterns remain unchanged from that of the parent ZIF-8 (Fig. S2b†), consistent with experimental observations. The larger experimental reduction in pore width for Tap-ZIF compared to the simulations likely reflects the additional framework flexibility that accompanies replacement of the heterocyclic MIm group with acetate.

In contrast to the Tap-ZIF results, Mel-ZIF shows reduced BET area with a value of 392 m^2^ g^−1^, suggesting either the formation of a new ZIF-like porous structure or the insertion of melamine molecules within the ZIF-8 pore. However, given the evidence of hydrogen bonding in Mel-ZIF observed by FT-IR, the latter is less likely. To further elucidate whether the amine groups of melamine are free or interacting *via* self-assembly with one another, CO_2_ sorption experiments were carried out, as amines are known binding sites for CO_2_.^66,67^ The CO_2_ uptake of the materials was measured at 290 K, 299 K and 308 K and up to 1 bar absolute pressure (Fig. S3 and S4a†). The isotherms were fitted using the Single Site Langmuir (SSL) model and the fitting parameters can be found in Table S1.† At 299 K and 1 bar, the CO_2_ uptake follows the trend: ZIF-8 (1.0 mmol g^−1^) > Tap-ZIF (0.7 mmol g^−1^) > Mel-ZIF (0.2 mmol g^−1^) (Fig. S4a†). The isotherms' linear shape suggests a physisorption mechanism. To examine the CO_2_ : N_2_ selectivity, we measured the N_2_ uptake of the materials at 299 K and up to 1 bar absolute pressure (Fig. S4b†). The derived SSL fitting parameters and fitted isotherms can be found in Table S1 and Fig. S5b,† respectively.

At these conditions, N_2_ uptake follows a similar trend: ZIF-8 (0.14 mmol g^−1^) > Tap-ZIF (0.1 mmol g^−1^) > Mel-ZIF (0.02 mmol g^−1^). Using the SSL fits for CO_2_ and N_2_ adsorption isotherms at 299 K, we calculated the competitive CO_2_ : N_2_ selectivity assuming 1 bar total pressure. The competitive selectivity *versus* the molar fraction of CO_2_ or N_2_ in a CO_2_/N_2_ stream (Fig. S5†) decreases with an increasing CO_2_ molar fraction; with the rate of decrease being higher for ZIF-8 compared to the constant behaviour of the modified samples. However, the average CO_2_ : N_2_ selectivity is not significantly affected and is similar between the three materials. Finally, to better understand the CO_2_ interactions with the materials, we derived the isosteric heat of adsorption (−Δ*H*_ads_) for each material (Table S2 and Fig. S6†). The results confirm that CO_2_ is physisorbed onto the materials, and that the heats of adsorption remain constant with varying CO_2_ loading, and similar between the materials.

Therefore, from this we suggest that in Mel-ZIF there are either: (i) not enough amino groups to modify the CO_2_ adsorption mechanism to chemisorption, or (ii) the existing –NH_2_ groups are not accessible, suggesting the interaction between neighbouring melamine molecules *via* H-bonding as supported by FT-IR and NMR. In the case of Tap-ZIF, while N_2_ sorption at 77 K shows an increase in BET area and a shift to smaller pores due to the presence of acetate defects, CO_2_ adsorption reveals a decreased uptake compared to ZIF-8. The narrower pores induced by acetate defects may hinder CO_2_ diffusion and reduce the available adsorption sites at room temperature, despite the increased BET area observed at cryogenic temperatures.

Given Tap is not taking part directly in the ZIF structure but facilitates the insertion of acetate defects, one can assume that Zn^2+^ is present mainly a tetrahedral ZnN_4_ coordination environment built with MIm linkers like in ZIF-8 with certain Zn–O coordination arising from acetate linkers (Fig. S1†).^68^ However, it remains unclear the role of Mel in the structure and whether it takes part as ligand coordinating to Zn^2+^. Therefore, the analysis of the Zn coordination environment and crystal structure focuses only on Mel-ZIF. To elucidate the relative ratio between the different components within Mel-ZIF, we carried out ^1^H-NMR experiments by dissolving Mel-ZIF in D-DMSO with a drop of 35% DCl,^42^ and compared it to the spectra of the individual components in D-DMSO, namely melamine, MIm and Zn acetate (Fig. S7–S9†). Mel-ZIF displays the signals from MIm, namely the one corresponding to the –CH_3_ at 2.55 ppm and a signal from the two aromatic protons C–H at 7.59 ppm (Fig. S10†). A signal from the –CH_3_ groups from acetate can also be observed at 1.89 ppm. Although melamine should not show ^1^H-NMR signals owing to the quick exchange of N–H_2_ protons from the amine groups, two signals are observed in the spectra of bare melamine arising from NH_3_^+^ as well as protonated sp^2^ nitrogen (N–H^+^) within the triazine probably due to the DCl solution not being 100% deuterated or from moisture (Fig. S7†). In Mel-ZIF (Fig. S10†), NH_3_^+^ signals are not observed, which is expected owing to the lower relative amount of melamine which allows the N–H_2_ labile protons to be exchanged by deuterium.

**Fig. 5:**
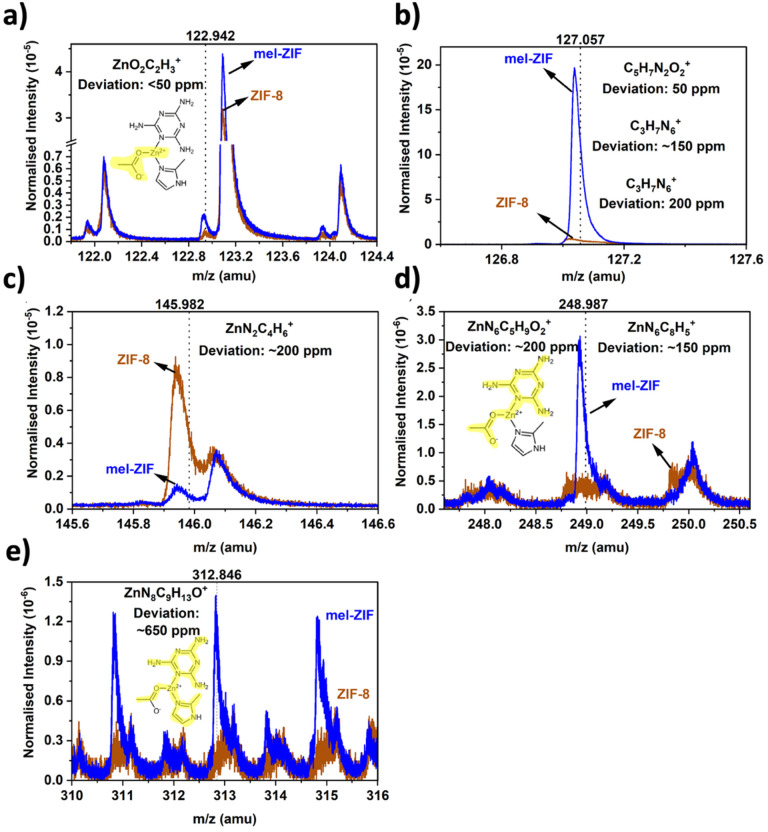
Regions of interest in the positive polarity where differences between Mel-ZIF and ZIF-8 are observed from ToF-SIMS at (a) 123 amu (b) 127 amu (c) 146 amu (d) 249 amu. (e) 313 amu. Possible fragments relating to identified peaks are displayed along with their deviation from the peak position.

However, an aromatic sp^2^ nitrogen (N–H^+^) signal can be observed at 7.83 ppm which was used to quantify the relative ratio between MIm, Mel and acetate, assuming that just one of the aromatic nitrogens is protonated. This yielded a calculated ratio of 1 : 1 : 1.85 MIm : Mel : Acetate. Due to its high mass resolution, ToF-SIMS was used to identify differences in molecular fragments between ZIF-8 and Mel-ZIF. An overview of the positive polarity mass spectrums is displayed in Fig. S11,† showing that normalised intensities are comparable across most of the *m*/*z*. Closer inspection shows at certain regions clear differences between the two samples. A higher intensity is observed at 122.942 amu in Mel-ZIF (Fig. 5), which is assigned to ZnO_2_C_2_H_3_^+^ (Fig. 5a), further confirming the presence of acetate ions. At 127.057 amu another peak is clearly distinguished for Mel-ZIF, which based on the smallest deviation from the peak is ascribed to either C_5_H_7_N_2_O_2_^+^ (Fig. 5b) or more likely the protonated melamine adduct C_5_H_7_N_6_^+^. Higher normalised intensity is found for ZIF-8 at 145.982, which is tentatively assigned to ZnN_2_C_4_H_6_^+^ (Fig. 5c), a Zn-MIm fragment.

A peak at 248.987 amu is observed in Mel-ZIF that could arise from ZnN_6_C_5_H_9_O_2_^+^ (Fig. 5d), resembling a Melamine coordinated to Zn acetate. Finally, a peak in Mel-ZIF at a high *m*/*z* around 312.846 amu could be based on ZnN_8_C_9_H_13_O^+^, derived from a Mel-Zn-MIm cluster similar to the one observed by Baslyman *et al.* when employing adenine.^18^ It is noted that the large deviation in Fig. 5e (∼650 ppm) likely arises due to the high amu of the fragment of interest being significantly outside of the calibration range. The ToF-SIMS results therefore suggest that Mel-ZIF incorporates an additional coordination environment around the Zn^2+^ centres comprised of acetate and melamine ligands, with a lower content of conventional Zn-MIm moieties.

**Fig. 6:**
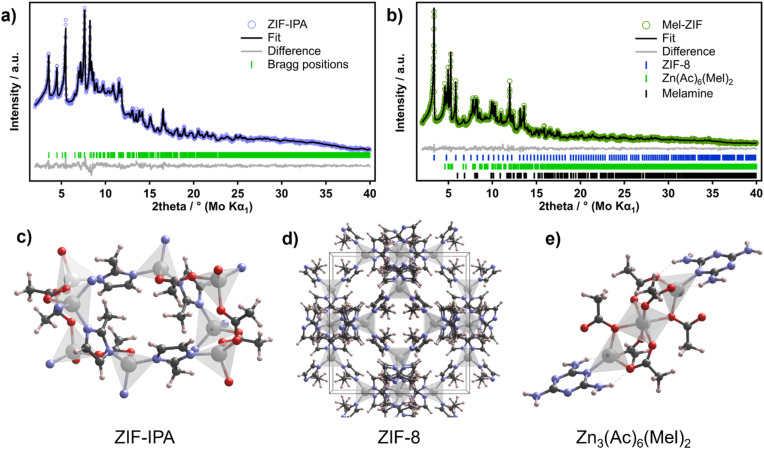
Rietveld refinement of the XRD pattern of (a) ZIF-IPA and (b) Mel-ZIF using monochromatic Mo Kα_1_ radiation (*λ* = 0.70926 Å). (c–e) Snapshots of the refined crystal structures of ZIF-IPA (c), ZIF-8 (b) and the Zn_3_(Ac)_6_(Mel)_2_ complex (e). Rietveld refinement details for ZIF-IPA: monoclinic(*P*2_1_/*c*), *a* = 11.980(2) Å, *b* = 14.831(2) Å, *c* = 9.9180(9) Å, *β* = 108.01(1)°. *R*_wp_ = 2.85%, *R*_exp_ = 2.04%, *χ*^2^ = 1.40. Rietveld refinement details for Mel-ZIF: ZIF-8 (16.6(2) wt%), cubic (*I*4̄3*m*), *a* = 17.016(2) Å; Zn(Ac)_6_(Mel)_2_ (35.1(4) wt%), triclinic (*P*1̄), *a* = 9.540(1) Å, *b* = 9.676(1) Å, *c* = 9.812(1) Å, *α* = 106.11(1)°,*β* = 101.64(1)°, *γ* = 113.70(1)°; melamine (48.3(6) wt%), monoclinic(*P*2_1_/*a*), *a* = 10.586(1) Å, *b* = 7.476(1) Å, *c* = 7.278(1) Å, *β* = 112.223(8)°. *R*_wp_ = 4.00%, *R*_exp_ = 3.76%, *χ*^2^ = 1.06. Colour code: Zn (silver), O (red), N (purple), C (grey), H (light pink).

Preliminary analysis of the powder XRD patterns of Mel-ZIF revealed the formation of ZIF-8 alongside an unidentified phase (Fig. 1a). To determine the different crystal structures present in Mel-ZIF, 3D electron diffraction (3D-ED) measurements were carried out (Table S3, see Methods for details†). Analysis of the resulting data revealed the presence of two distinct crystalline phases: the parent ZIF-8 framework and a Zn-acetate-melamine coordination compound (Zn_3_(Ac)_6_(Mel)_2_). The latter, which to our knowledge is unreported to date, consists of a central Zn^2+^ ion in an octahedral coordination environment connected to two Zn^2+^ ions in a tetrahedral coordination geometry by six bridging acetate ligands and terminal Mel ligand completing the coordination sphere of each terminal Zn ion (Fig. 6e). These findings confirm that the introduction of melamine and acetate leads to partial disruption of the formation of the ZIF-8 framework with competing formation of additional phases. Analysis of the powder XRD data *via* Rietveld refinement to quantify the relative abundance of the different phases yielded the presence of a third crystalline phase that was identified as recrystallized melamine. The refinement resulted in relative abundances of 16.6(2)% for ZIF-8, 35.1(4)% for Zn(Ac)_6_(Mel)_2_ and 48.3(6)% for crystalline melamine (Fig. 6b, d and e). The presence of several phases supports the conclusion that melamine participates both in coordination with Zn^2+^ centres (as observed *via* FTIR, NMR and ToF-SIMS). However, it fails to take part in the ZIF-8 structure or in additional supramolecular frameworks.

The discrepancy in the relative abundances found *via* Rietveld analysis compared to the molar ratio obtained *via*^1^H-NMR likely arises from the difficulty of accurately detecting melamine in the NMR experiment where some fragments may not be protonated and the –NH_2_ are quickly exchanged by deuterium. Increasing the melamine content in the synthesis to 2 and 5 equivalents in Mel-ZIF just resulted in the formation of more crystallized melamine as quantified by Rietveld analysis of XRD data (Fig. S12–14 and Table S4†). Additionally, ZIF-8 was synthesized under the same conditions as Mel and Tap-ZIF (using Zn^2+^ acetate as Zn source, in IPA at 70 °C), albeit in the absence of the N-heterocycle and interestingly, due to the slow nucleation promoted by the acetate ligands, another unreported crystal structure was obtained that did not match the powder XRD pattern of ZIF-8 (Fig. S15†). This structure, labelled ZIF-IPA, was solved by 3D-ED. ZIF-IPA crystallises in the monoclinic space group *P*2_1_/*c* with cell parameters *a* = 11.250(4) Å, *b* = 14.7993(15) Å, *c* = 9.9128(11) Å, *β* = 98.606(19)° (ZIF-IPA-1). The structure consists of two crystallographically independent Zn ions interconnected by bridging acetate and imidazolate linkers and having two distinct tetrahedral coordination environments, ZnN_3_O and ZnNO_3_ (Fig. 6a and c).

This network of interconnected ZnN_3_O and ZnNO_3_ tetrahedra extends along the *c* and *b* axes forming corrugated layers which stack parallel to the *a* axis (Fig. S16†). Interestingly, a closely related polymorph of this material was also found in the sample with only slightly different unit cell parameters: *a* = 11.841(5) Å, *b* = 14.8344(17) Å, *c* = 9.9080(18) Å, *β* = 107.92(3)° (space group *P*2_1_/*c*, ZIF-IPA-2). A closer inspection to the structure of these polymorphs reveals that the difference between them lies differ in the relative orientation of the imidazole and acetate linkers in the structure (Fig. S16†). The effect of this difference in the linker orientation is clearly seen when comparing the view of both structures along the *a*-axis showing that, while ZIF-IPA-1 displays an ‘open-pore’ structure with nanoporous rectangular channels of 3.9 × 5.3 Å, ZIF-IPA-2 shows instead a ‘closed-pore’ conformation with the imidazole linkers blocking the channels (Fig. S17†). Notably, only ZIF-IPA-2 polymorph was observed in the Rietveld refinement of the PXRD data (Fig. 6a), possibly due to the evaporation of occluded solvent molecules or under the vacuum of the electron diffraction experiment, also indicating a certain degree of structural flexibility in ZIF-IPA along the *bc* plane. Guo *et al.* observed a similar structure when employing Zn^2+^ acetate in the ZIF-8 synthesis in dimethylsulfoxide, where acetate linkers linked Zn atoms to form a 1D zigzag chain, which then generate a 3D framework.^68^ Interestingly, this structure contradicts previous findings by Schneider and co-workers that observed that the utilization of Zn acetate led to ZIF-8 structures with slightly higher particle sizes compared to other salts.^11^ Therefore, the difference in crystal structure and nucleation could be due to reaction temperature (70 °C *vs.* room temperature), solvent (IPA *vs.* MeOH), or MIm-Zn acetate-solvent ratio, however the elucidation of such differences are beyond the scope of this work.

With all data in hand, we can confirm that while Mel coordinates Zn acetate moieties, Tap promotes the synthesis of a defective ZIF-8, which is not formed in its absence. The differences in reactivity stems from the subtle differences in the heterocyclic ring: triazine in the case of Mel and pyrimidine in the case of Tap. The additional C–H bond in Tap reduces the electron withdrawal from the ring, resulting in a higher p*K*_a_ compared to Mel (6.8 *vs.* 5.0),^69^ and higher basicity.^70^ Consequently, Tap is more likely to deprotonate MIm ligands, facilitating the synthesis of a defective ZIF-8. Protonated Tap is less available for Zn^2+^ coordination and likely displays higher solubility in the reaction media, as observed in other protonated heterocyclic amines.^71^ As a result, Tap remains in the solution phase and is not incorporated in the framework, consistent with its absence in the final materials. In contrast, Mel is less basic and cannot efficiently deprotonate MIm. Instead, it coordinates to Zn^2+^*via* the heterocyclic ring and establishes supramolecular interactions with the acetate anions. Consequently, just a small amount of ZIF-8 is formed in the Mel system, as observed *via* Rietveld refinements.

Next we report the thermal conversion of the prepared ZIF-based materials into nitrogen-doped carbon to elucidate the impact of the morphology and crystal structure on parameters such as pore structure, nitrogen functionalities and graphitic nature. The thermal treatment of ZIF-8 at temperatures above 700 °C results in the degradation of the MIm imidazole ligands and their conversion into microporous nitrogen-doped carbons, with the Zn^2+^ sites shifting from a tetrahedral ZnN_4_ coordination (in the case of the parent ZIF-8) to a planar porphyrin-like ZnN_4_ coordination.^72^ ZIF-8 derived carbon materials have therefore been widely studied in diverse fields of electrocatalysis and energy storage, where the electronic conductivity of the active material, which is often determined by the pyrolysis temperature, is crucial. We therefore selected 1000 °C as the pyrolysis temperature to assess the impact of the different ZIF-8 structures on the resulting carbon materials properties, as such temperature results in an efficient electronic conductivity suitable for energy-related applications.^73,74^ Thermogravimetric analysis (TGA) performed under N_2_ atmosphere (Fig. S18a†) confirms that the presence of melamine phases results in a substantially lower pyrolysis yield. The initial 35 wt% drop at 300 °C could be correlated to the degradation of melamine, (40.94% relative abundance as observed by Rietveld refinement) as melamine adducts usually form carbon nitride-like polymers which degrade at temperatures above 700 °C.^75,76^ ZIF-8 and Tap-ZIF, however, display a similar degradation profile. TGA under air reveals subtle differences in the thermal stability of ZIF-8 and Tap-ZIF (Fig. S18b and c†). ZIF-8 exhibits a minor weight loss at around 300 °C, likely corresponding to the early decomposition of MIm ligands.^77^ In contrast, Tap-ZIF shows no such feature, suggesting a framework less prone to early decomposition and in agreement with the higher thermal stability of acetate ligands compared to MIm.^78^ The main decomposition at 500 °C appears sharper for Tap-ZIF, reflecting a higher defect concentration that leads to a more abrupt framework collapse. These thermal features support a defect-modified structure in Tap-ZIF, consistent with differences in the pore structure, gas uptake and morphology while maintaining identical XRD patterns.

**Fig. 7:**
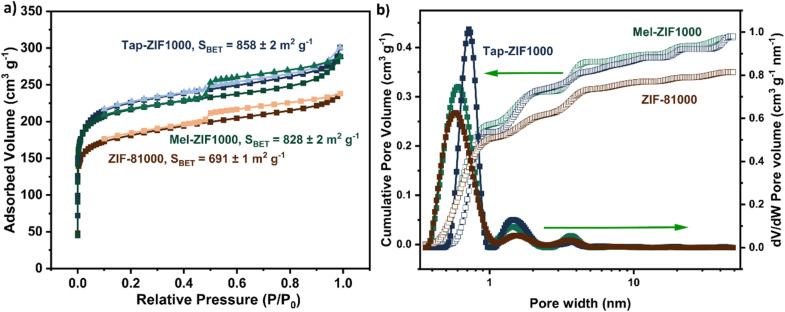
N_2_ sorption isotherms with specific BET areas of ZIF-derived materials pyrolized at 1000 °C (a). (b) Pore size distributions and cumulative pore volumes calculated using 2D-non-local density functional theory (NLDFT) heterogeneous surface carbon model in SAIEUS software of carbonized materials.

BET measurements confirm that the presence of mixed phases, as well as defective ZIF structures strongly impacts the pore structure of the resulting nitrogen-doped carbons. According to N_2_ sorption measurements at 77 K, both Mel-ZIF1000 and Tap-ZIF1000 exhibit a higher BET surface area (828 ± 2 m^2^ g^−1^ and 858 ± 2 m^2^ g^−1^, respectively) than ZIF-81000 (691 ± 1 m^2^ g^−1^), which contrasts with the trend observed in the precursor materials (Fig. 7a). All three carbonized samples display isotherms characteristic of Type I behaviour, indicative of the presence of mostly micropores. Pore size distribution analysis of the carbonized samples reveals a predominantly microporous structure, with emerging mesoporosity on Mel-ZIF1000 and Tap-ZIF1000 (Fig. 7b). The carbonized materials show primary micropores at 0.58, 0.61 and 0.72 nm, for ZIF-81000, Mel-ZIF1000 and Tap-ZIF1000 respectively, with Tap-ZIF1000 showing a narrower micropore size distribution. Peaks can also be observed at 1.5 nm and a weak mesoporous contribution near 3.6 nm. Both Mel-ZIF1000 and Tap-ZIF1000 display higher mesoporosity compared to ZIF81000, achieving nearly a 30% higher mesopore volume of 0.12 cm^3^ g^−1^ (*vs.* 0.09 cm^3^ g^−1^) for ZIF-81000 (Table S5†). The increase in mesoporosity may arise from the release of low molecular weight cyanides as well as ammonia from melamine decomposition at low temperatures (in the case of Mel-ZIF1000),^79^ which generate bigger pores in the carbon framework, and from the presence of acetate defects, in the case of Tap-ZIF1000. Powder XRD patterns (Fig. S20†) of the carbon-based materials show two broad reflections centred at 23° and 43° 2*θ*, which can be assigned to the (002) and (101) planes of turbostatic disordered carbon, respectively.^80,81^ This indicates the formation of largely amorphous carbon structures following pyrolysis at 1000 °C. Additionally, no sharp diffraction peaks characteristic of crystalline ZnO are observed, suggesting that the residual Zn species are either coordinated to nitrogen functionalities,^8,72^ or present as fine amorphous domains below the detection limit of the experiment.

Raman spectroscopy further confirms the defective nature of the carbon materials. All three pyrolyzed samples show two broad peaks at approximately 1340 and 1585 cm^−1^ in the first-order spectra, corresponding to the D (disorder-induced) and G (graphitic) bands respectively (Fig. S20a†). The intensity ratio of the D and G bands (*I*_D_/*I*_G_) is often used as an indicator for the degree of disorder in carbon materials. The N-doped carbons derived from Tap-ZIF, Mel-ZIF and ZIF-8 display similarly high average *I*_D_/*I*_G_ values (1.59, 1.33, and 1.41, respectively), along with a weak second-order spectrum (2D band), consistent with the formation of disordered carbon frameworks composed of nanocrystalline graphitic domains (Fig. S20b–d†). Tap-ZIF1000 exhibits the highest *I*_D_/*I*_G_ (1.59), which may reflect a higher degree of structural disorder. This trend is consistent with the presence of acetate-induced defects in the precursor framework, likely facilitating defect formation upon carbonization. In contrast, Mel-ZIF1000 shows the lowest *I*_D_/*I*_G_ value (1.33), suggesting that melamine-derived decomposition products may promote the formation of more ordered graphitic domains during pyrolysis.^82^ While the absolute differences in *I*_D_/*I*_G_ are moderate, they support a trend consistent with the distinct chemical environments introduced by melamine and Tap. Finally, X-ray photoelectron spectroscopy (XPS) confirms the formation of zinc–nitrogen–carbon (Zn–NC) materials with Zn contents of ∼14 wt% in the case of ZIF-81000 and Mel-ZIF1000 and 20.6 wt% in the case of Tap-ZIF1000 (Table S6†) which agrees with a lower theoretical organic-to-metal ratio of Tap-ZIF compared to ZIF-8 arising from the acetate defects. While Zn displays a boiling point of 907 °C, the stabilization of Zn^2+^ species by nitrogen moieties results in the formation of porphyrin-like ZnN_4_ sites preventing its complete evaporation.^72,83^ However, the high Zn wt% value compared to literature suggests the presence of residual surface ZnO fragments and may differ to the bulk composition of the ZIF-derived carbons.^84,85^ The high resolution N 1s spectra of the materials shows very similar profiles; four main contributions are observed that stand for pyridinic, Zn–N, pyrrolic and graphitic nitrogen environments (Fig. S21†).^86,87^ Both Mel-ZIF and Tap-ZIF display a substantially larger pyridinic contribution (at 398.3 eV) compared to ZIF-81000 (Table S7†), which instead shows a larger Zn–N binding energy (at 399.5 eV) suggesting the formation of more nitrogen-coordinated Zn sites. C 1s spectra however reveals minimal differences within the materials (Fig. S22†). Meanwhile high-resolution Zn 2p spectra for Tap-ZIF1000 (Fig. S23†) shows a slight 0.2 eV shift of the Zn 2p^3^ binding energy from 1021.88 to 1021.68 eV which could imply mild differences in its local coordination environment arising from the acetate defects present in Tap-ZIF. Along with BET, XPS and Raman confirmed that both the phase mixture present in Mel-ZIF as well as the defects observed in Tap-ZIF modify the pore structure, the carbon nanostructure and the chemical composition of the resulting Zn–NC materials. Due to the early degradation of melamine-based structures,^88^ the volatile products induce mesopore volume in the carbon framework and result in lower Zn content and a more graphitic nature, while the acetate defects induced by Tap result in a slightly higher micropore volume, defective carbon structure and an altered Zn electronic state, as observed by the shift in the Zn 2p^3^ binding energy.

## Conclusions

In summary, in this work we report the utilization of the heterocyclic amines melamine and 2,4,6-triaminopyrimidine to modulate the structure of ZIF-8 and subsequently correlate such structural changes with the properties of the Zn–NCs obtained by high temperature pyrolysis. Through a combination of advanced characterization techniques and theoretical calculations, we reveal that melamine coordinates with Zn^2+^ acetate to form a previously unreported complex, Zn_3_(Ac)_6_(Mel)_2_, resulting in a mixture of phases including ZIF-8. In contrast, Tap promotes the formation of a defective ZIF-8 containing residual acetate ligands, as proved by theory and experiment, which results in a decrease in pore size while increasing the specific BET area. In the absence of any heterocyclic amine, a new flexible crystalline phase emerges featuring two distinct Zn^2+^ tetrahedral environments bridged by acetate and imidazolate linkers, a structure not previously reported and exhibiting subtle polymorphism, potentially *via* unresolved desolvation. Upon pyrolysis, all modified ZIFs yield Zn–NC materials with enhanced BET surface area, underlining the crucial role of precursor structure in determining the final carbon architecture. This work highlights a versatile synthetic strategy for incorporating N-heterocyclic amines into ZIF chemistry, offering new avenues for designing functional porous crystalline materials for gas separations and carbon-based scaffolds for electrochemical technologies such as oxygen and CO_2_ electroreduction.

## Author contributions

J. C.-G. co-led the study, performed the powder X-ray diffraction analysis, led the electron diffraction proposal and contributed to the manuscript writing; J. Z. performed the thermal treatment, BET measurements, XRD measurements, and SEM measurements; I. I. performed CO_2_ sorption measurements; R. H. performed TGA and Raman measurements; J. P. T. performed the electron diffraction experiments and data processing; A. P. performed TOF-SIMS measurements; E. S. performed XPS measurements; H. T. performed NMR measurements; E. H. W. performed the calculations on Tap-ZIF; C. P. provided supervision, funding and assisted in manuscript preparation; J. B. conceptualized the work, led the work, synthesized the ZIF materials and wrote the initial draft. All authors contributed to the final draft of the manuscript.

## Conflicts of interest

There are no conflicts to declare.

## Supplementary Material

TA-013-D5TA04831A-s001

## Data Availability

The data supporting our findings are available on the Zenodo research data repository (https://zenodo.org/records/16261501).
