# Supplementary material for: Impact of N-heterocyclic amine modulators on the structure and thermal conversion of a zeolitic imidazole framework
Source: J Mater Chem A Mater. 2025 Jul 24;13(34):28006–18. doi: 10.1039/d5ta04831a (PMC12311777; doi:10.1039/d5ta04831a)
Supplement: TA-013-D5TA04831A-s001 [file TA-013-D5TA04831A-s001.pdf]

Supplementary Information (SI) for Journal of Materials Chemistry A.
This journal is © The Royal Society of Chemistry 2025

Supporting information for:

Impact of *N*-heterocyclic amine modulators on the structure and thermal conversion of a zeolitic imidazole framework

Javier Castells-Gil,*‡[a] Jinjie Zhu, ‡[b] Ioanna Itskou,[b] Emma H. Wolpert,[c] Robert D. Hunter,[b] Jeremiah P. Tidey,[d] Angus Pedersen, [e] Elisa Solvay, [b] Helen Tyrrell, [b] Camille Petit [b] and Jesús Barrio*[b]

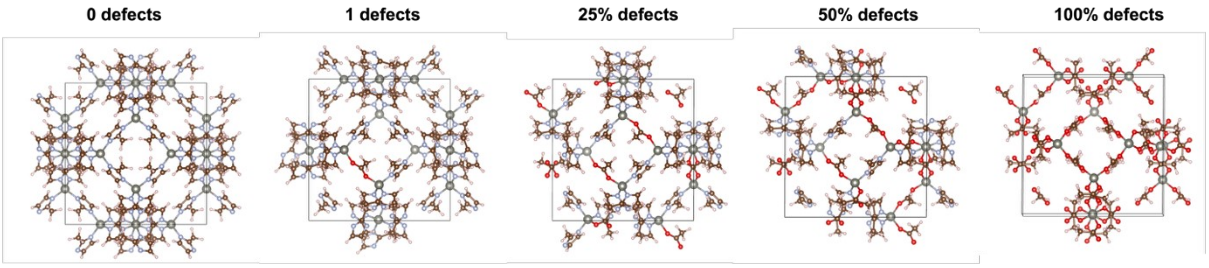

Figure S1. Structure of ZIF-8 with different amounts of acetate defects.

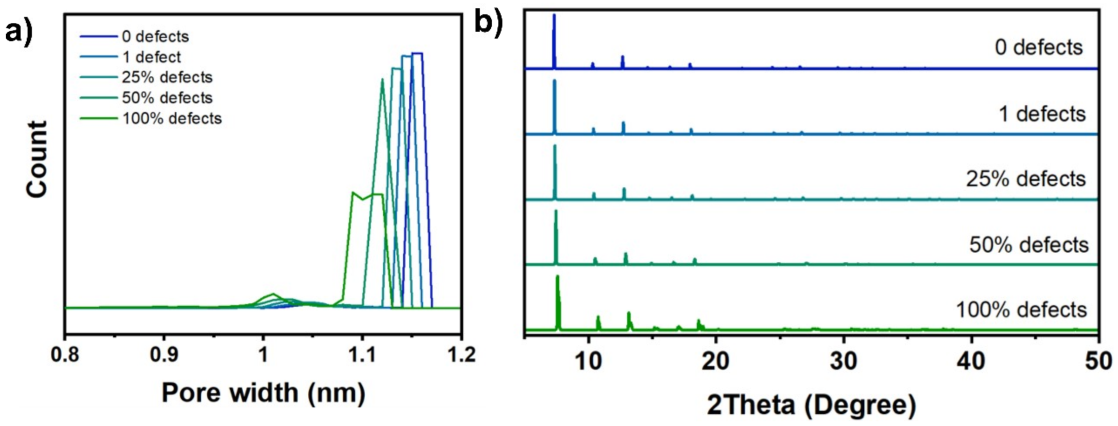

Figure S2. Simulated pore size distribution (a) and XRD patterns (b) of ZIF-8 with different defect concentrations

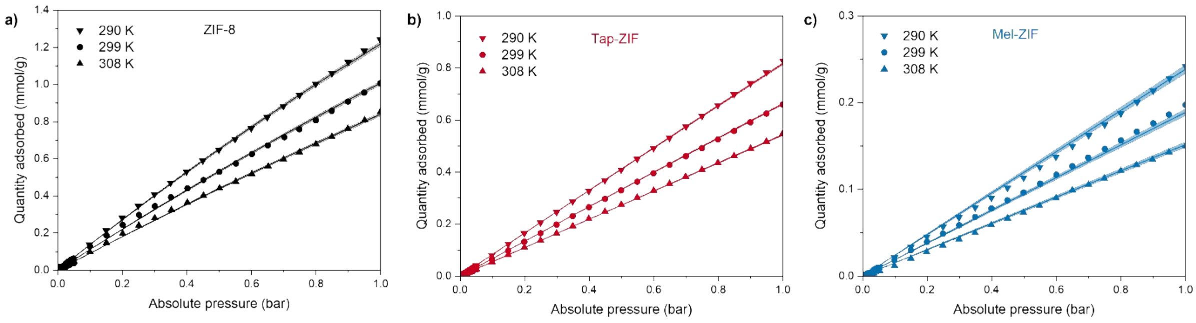

Figure S3. CO_2 adsorption isotherms (290, 299, 308 K), fitted using the SSL model for a) ZIF-8, b) Tap-ZIF and c) Mel-ZIF. Symbols represent experimental data points, solid lines represent the isotherm fit, and shaded areas represent confidence bounds.

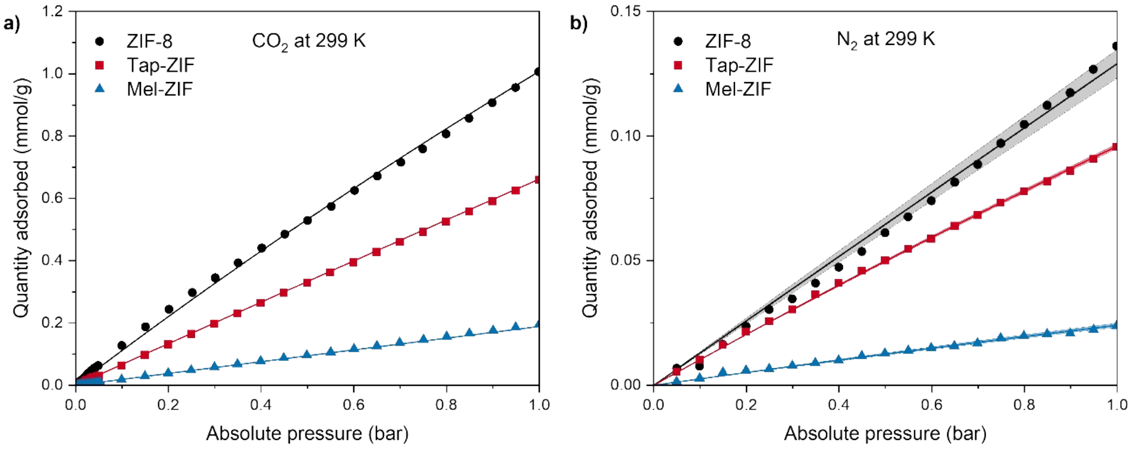

Figure S4. Experimental a) CO_2 and b) N_2 adsorption isotherms of ZIF-8, Tap-ZIF and Mel-ZIF at 299 K, fitted using the single site Langmuir (SSL) model. Symbols represent experimental data points, and solid line represents the isotherm fit. Shaded areas in b) represent confidence bounds.

Table S1. SSL coefficients, as derived from fitting experimental N_2 (299 K) and CO_2 (290, 299, 308 K) adsorption data. The values in parentheses represent uncertainty values.

Gas	SSL coefficient	ZIF-8	Tap-ZIF	Mel-ZIF
N_2	q_s (mol kg^{-1})	50.00 (1.22)	1.30 (7.80×10^{-3})	0.33 (6.47×10^{-3})
	b_0 (bar^{-1})	2.59×10^{-3} (6.35×10^{-5})	8.00×10^{-2} (5.11×10^{-4})	8.00×10^{-2} (1.69×10^{-3})
CO_2	q_s (mol kg^{-1})	9.32 (0.05)	50.00 (0.11)	30.68 (0.28)
	b_0 (bar^{-1})	1.17×10^{-4} (6.35×10^{-7})	1.41×10^{-5} (3.20*10^{-8})	3.20×10^{-6} (2.88×10^{-8})
	-ΔU (J mol^{-1})	17258 (13.48)	17058 (5.65)	18815 (22.39)

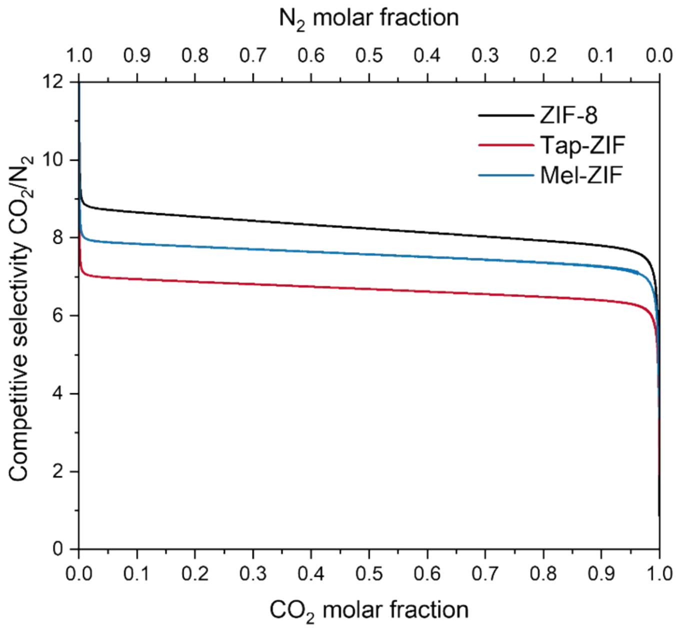

Figure S5. Competitive CO_2:N_2 selectivity for ZIF-8, TAP-ZIF and Mel-ZIF over ranging CO_2 or N_2 molar fraction in the CO_2/N_2 stream (299 K, 1 bar). The CO_2:N_2 selectivity was calculated by fitting experimental CO_2 and N_2 adsorption data obtained at 299 K for all materials to the SSL model.

Table S2. Heat of adsorption values for ZIF-8, Tap-ZIF and Mel-ZIF, as derived from the SSL fit on experimental CO_2 adsorption data (290, 299, 308 K).

**Sample**	**$-\Delta H_{ads}$ (kJ mol^{-1})**
ZIF-8	17
Tap-ZIF	17
Mel-ZIF	19

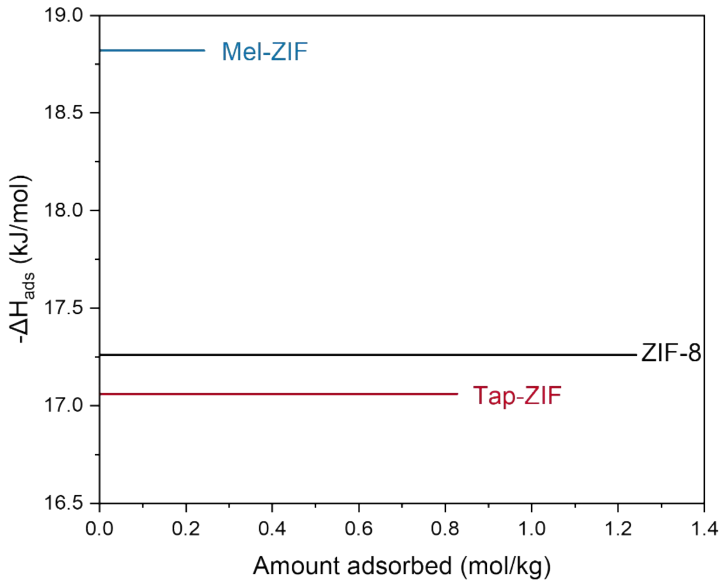

Figure S6. Isosteric heat of adsorption of ZIF-8, Tap-ZIF, and Mel-ZIF plotted against CO_2 loading.

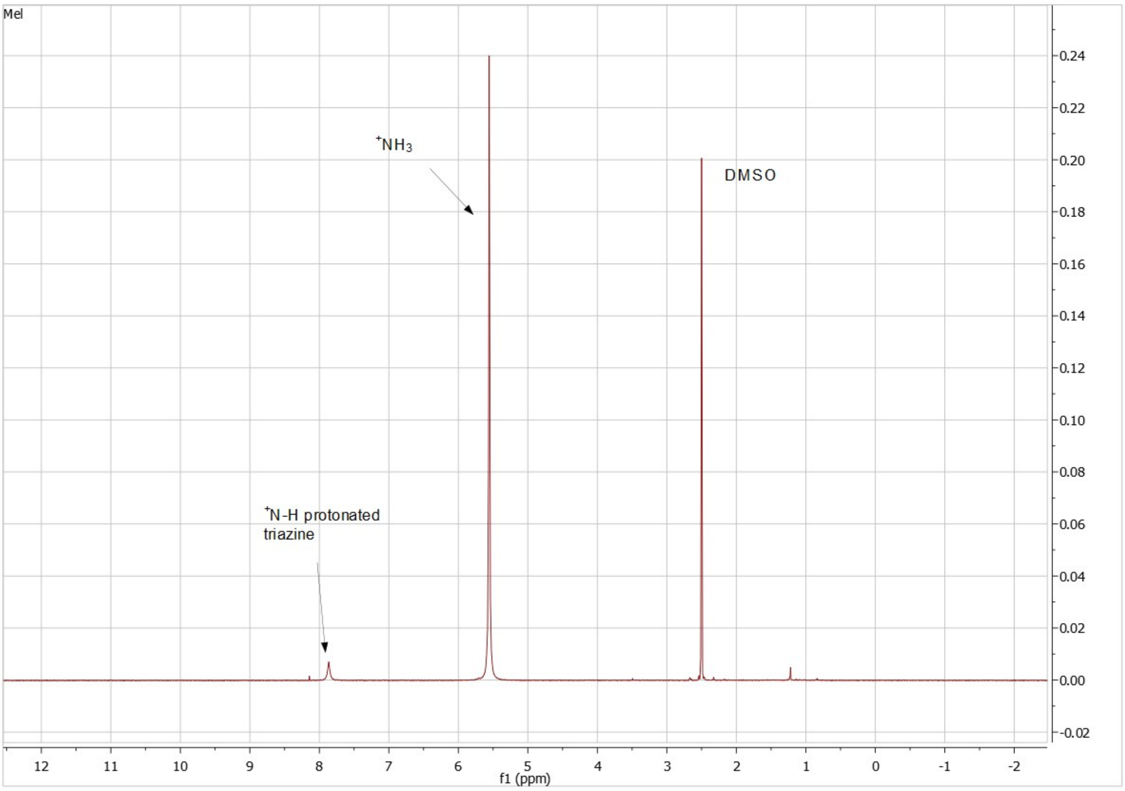

Figure S7. ^{1}H-NMR spectra of melamine. While melamine should not display 1H-NMR signals due to the quick exchange of $-NH_2$ protons with deuterium, two signals are observed that corresponds to NH_3^+ as well as protonated sp^2 nitrogen within the triazine (7.89 ppm).

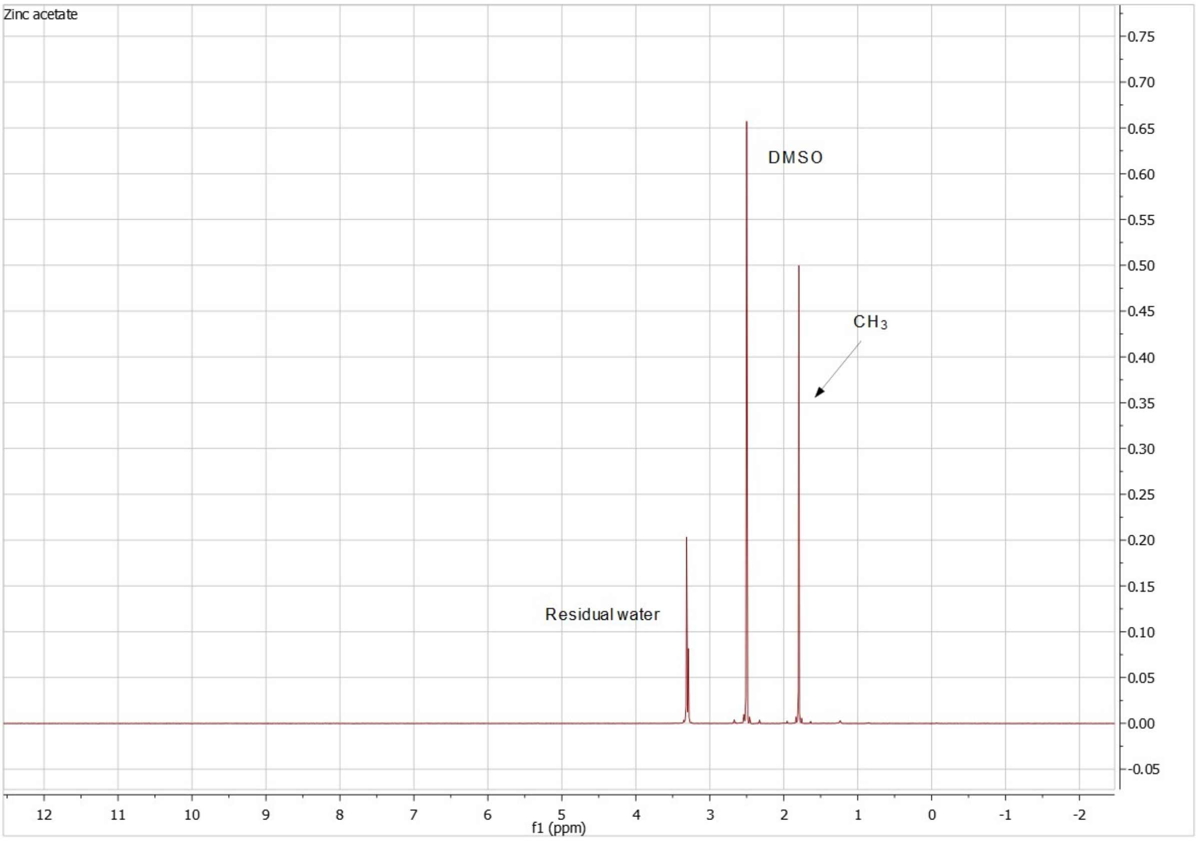

Figure S8. ^{1}H-NMR spectra of Zn acetate dihydrate.

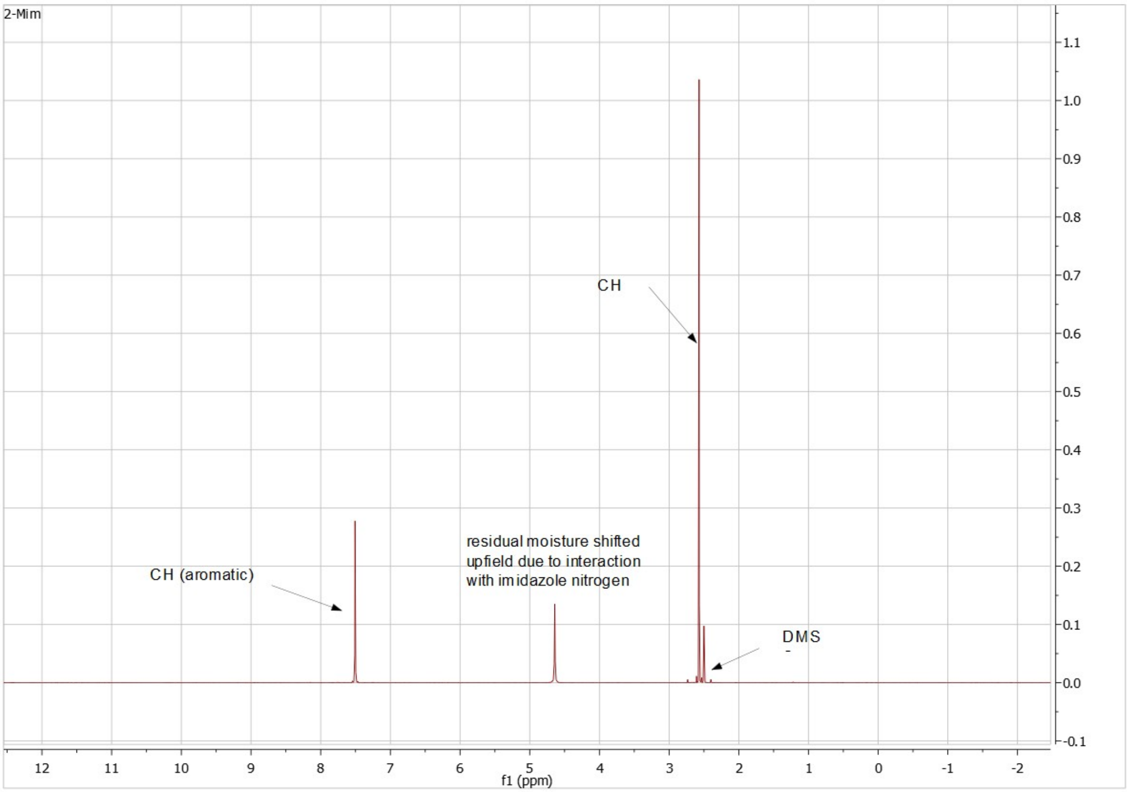

Figure S9. ^{1}H-NMR spectra of 2-methyl imidazole.

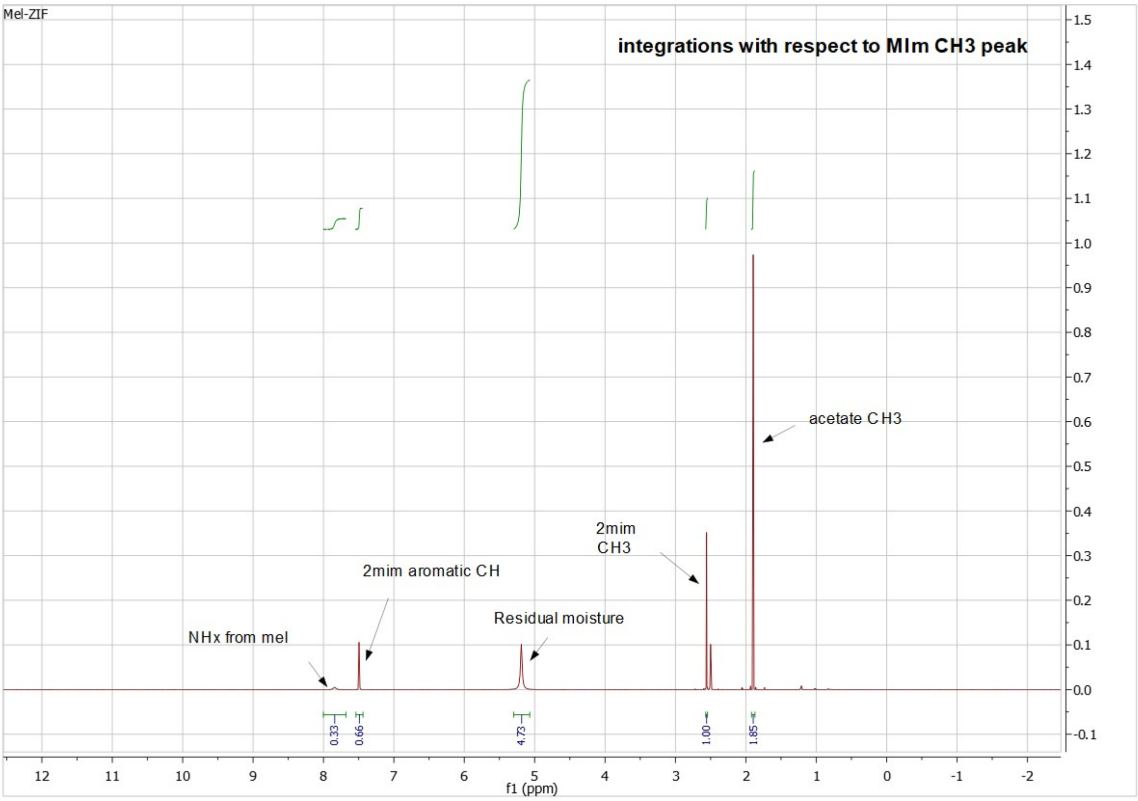

Figure S10. ^{1}H-NMR spectra of Mel-ZIF and integrations of each signal with respect to the CH_3 peak of Mim. The spectra display the signals from methyl imidazole ($-CH_3$, 2.55 ppm), and a signal from the two aromatic C-H protons (7.59 ppm). The signal from the CH_3 groups within acetate can also be observed (1.89 ppm).

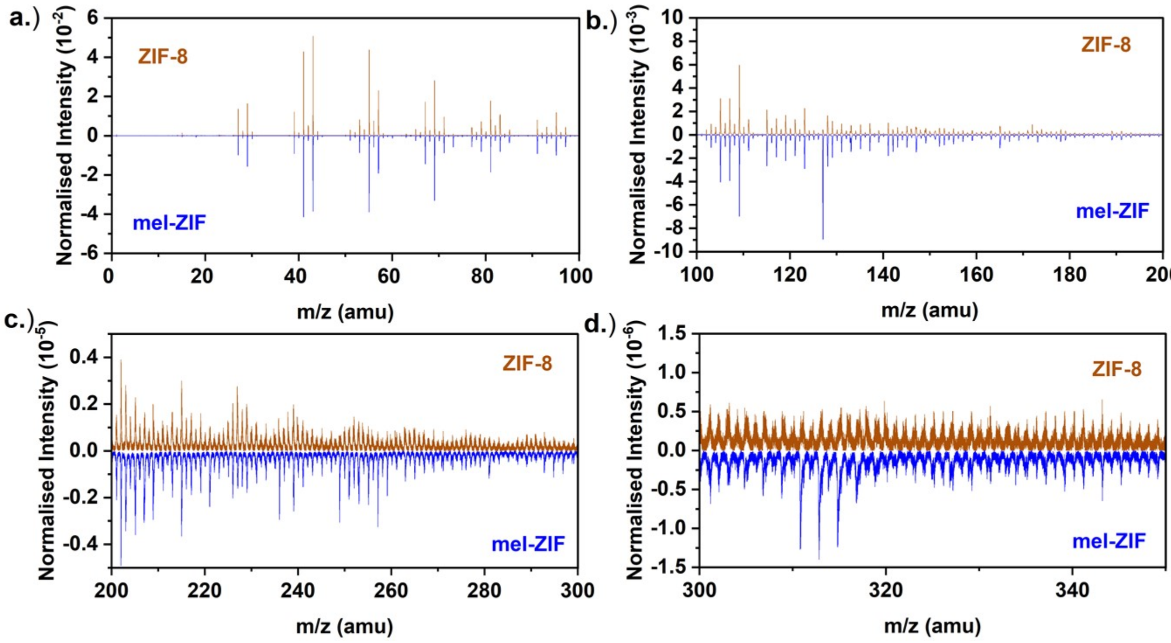

Figure S11. ToF-SIMS in the positive polarity normalised intensity to total counts comparison for ZIF-8 and Mel-ZIF from a) 0-100 amu. b) 100-200 amu. c) 200-300 amu. d) 300-350 amu. Mel-ZIF was normalised in the negative axis was comparison.

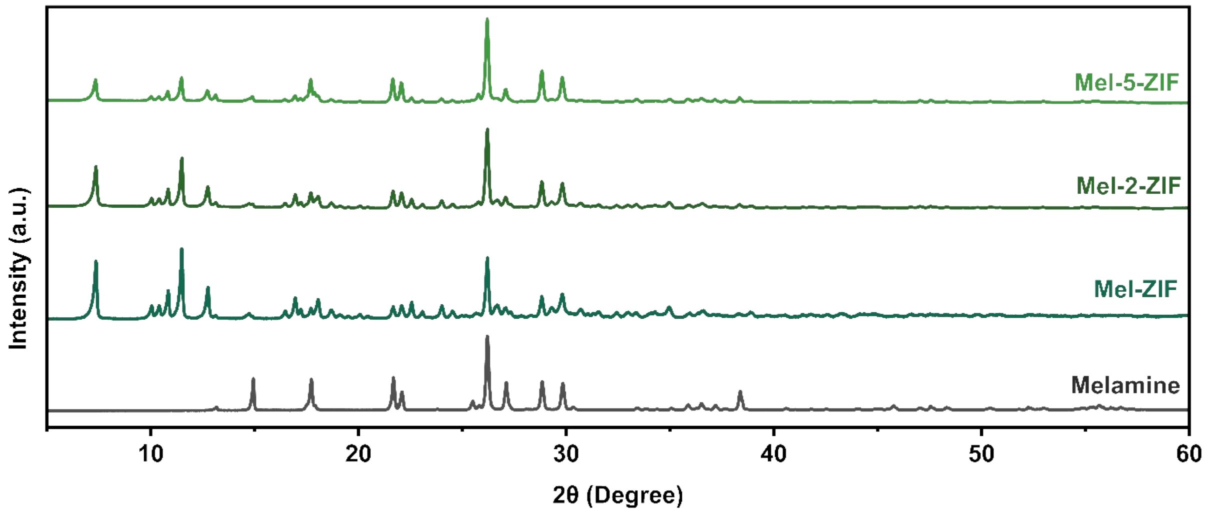

Figure S12. X-ray diffraction pattern of Mel-ZIF synthesized with different melamine contents.

Table S3. Experimental crystallographic details of single crystal 3D electron diffraction data.

	Mel-ZIF_aP	***ZIF-8***	***ZIF-IPA_B98 (ZIF-IPA-1)***	***ZIF-IPA_B108 (ZIF-IPA-2)***
**Crystal data**				
Chemical formula	$C_{18}H_{30}N_{12}O_{12}Zn_3$	$C_8H_{10}N_4Zn$	$C_{12}H_{16}N_4O_4Zn_2$	$C_{12}H_{16}N_4O_4Zn_2$
M_r	802.68	227.59	411.06	411.06
Crystal system, space group	Triclinic, *P*-1	Cubic, *I*-43*m*	Monoclinic, $P2_1/c$	Monoclinic, $P2_1/c$
Temperature (K)	100(5)	100(5)	125(5)	125(5)
a, *b*, *c* (Å)	9.5374(5)<br>9.5890(6)<br>9.7833(4)	17.0198(13)<br>17.0198(13)<br>17.0198 (13)	11.250(4)<br>14.7933(15)<br>9.9128(11)	11.841(5)<br>14.8344(17)<br>9.9080(18)
α, β, γ (°)	105.707(5)<br>101.723 (4)<br>113.885(6)	90<br>90<br>90	90<br>98.606(19)<br>90	90<br>107.92(3)<br>90
V (Å^3)	736.15(9)	4930.2(7)	1631.1(6)	1655.9(8)
Z	1	12	4	4
Radiation type	Electron, λ = 0.02510 Å			
**Data collection**				
Scan range (°)	See _diffrn_measurement_details in CIFs for individual component angular ranges.			
measured, independent, observed [$I \geq 2u(I)$] reflections	58932<br>6374<br>4736	59057<br>832<br>703	32154<br>2952<br>1776	9732<br>2605<br>1583
R_{int}	0.245	0.535	0.298	0.165
$(\sin \theta/\lambda)_{max}$ (Å^{-1})	0.834	0.598	0.626	0.626
**Refinement**				
R_1, wR_2 ([$F^2 > 2\sigma(F^2)$])	0.1658, 0.3497	0.1251, 0.2862	0.1386, 0.3539	0.1280, 0.3428
R_1, wR_2 (all)	0.2003, 0.3671	0.1362, 0.2965	0.1953, 0.3861	0.1768, 0.3788
GoF(*S*) (inc., excl. restraints)	1.0295, 1.0462	1.0437, 1.0437	1.2108, 1.2147	1.0552, 1.0591
No. reflections	6374	832	2952	2605
No. parameters	229	35	204	203
No. restraints	201	0	18	18
$\Delta\varphi_{max}$, $\Delta\varphi_{min}$ (as reported by Olex2.refine, AC07-014)	1.31, −1.02	0.94, −0.78	0.86, −0.73	1.49, −0.85

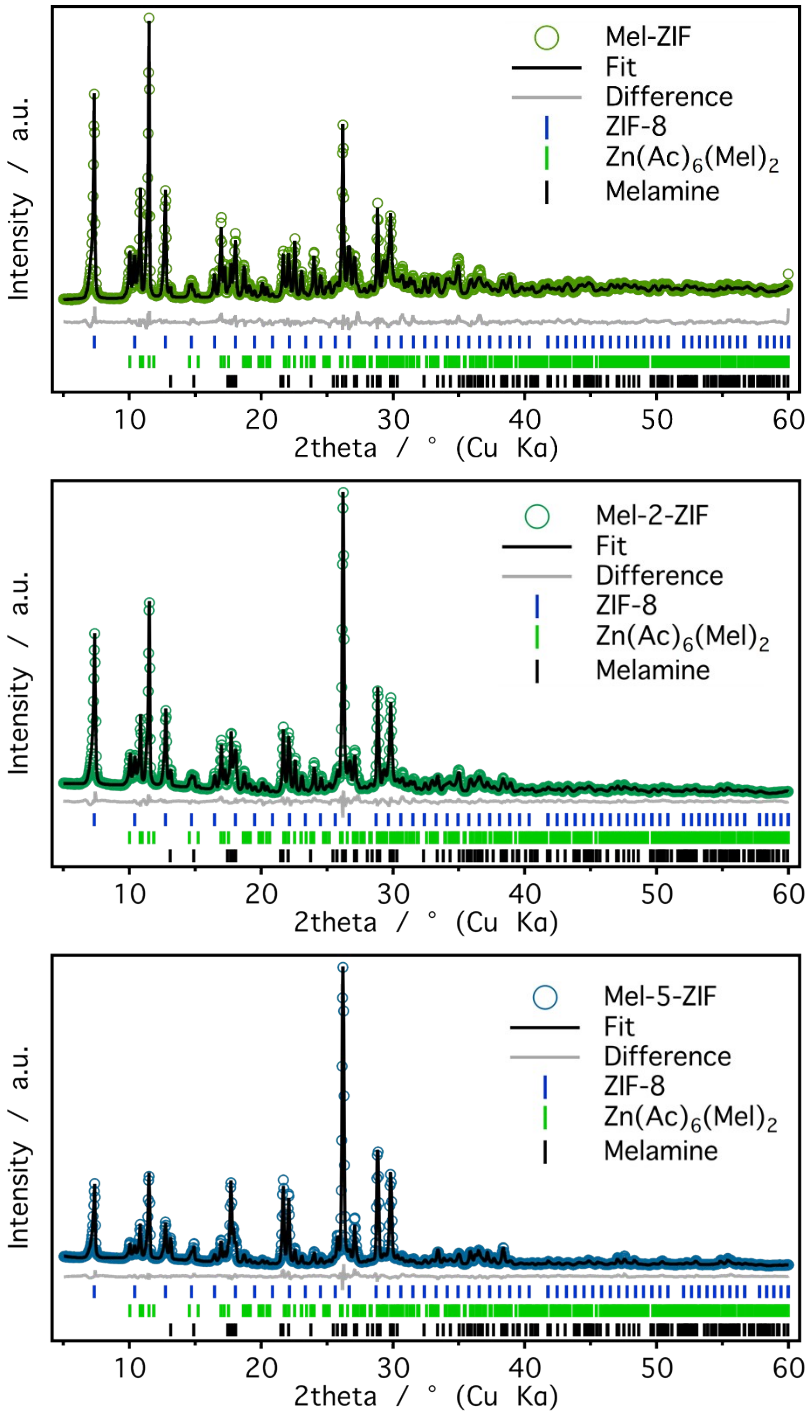

Figure S13. Rietveld refinement plots of Mel-ZIF (top), Mel-2-ZIF (middle) and Mel-5-ZIF (bottom) measured with Cu Kα radiation. Rietveld refinement details in table S3

Table S4 – Relative weight fractions and agreement factors from the Rietveld refinements of Mel-ZIF, Mel-2-ZIF and Mel-5-ZIF.

	**ZIF-8 Wt. %**	**$Zn(Ac)_6(Mel)_2$ Wt. %**	**Melamine Wt. %**	**Rwp %**	**GoF**
**Mel-ZIF**	16.4(4)	41.4(5)	42.2(5)	11.56	3.59
**Mel-2-ZIF**	9.7(1)	27.8(5)	62.5(5)	7.00	6.73
**Mel-5-ZIF**	4.8(1)	9.7(1)	81.4(3)	6.41	4.55

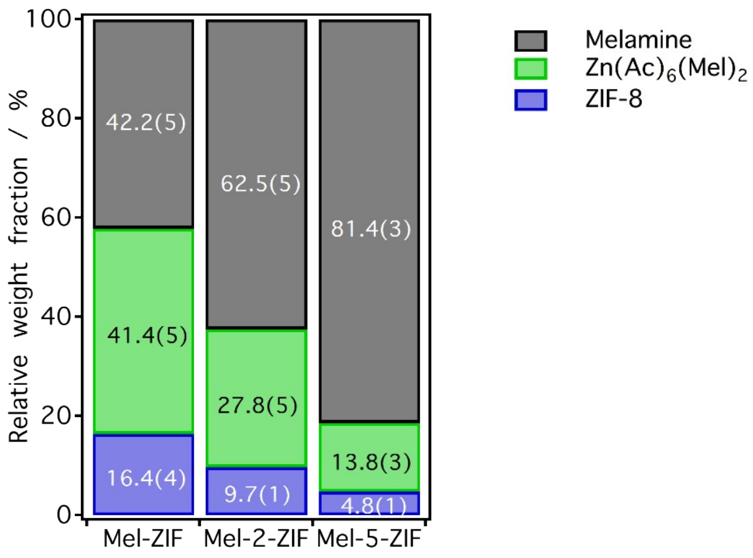

Figure S14. Relative weight fractions for each phase as obtained from the Rietveld refinements of Mel-ZIF, Mel-2-ZIF and Mel-5-ZIF samples in Figure S13.

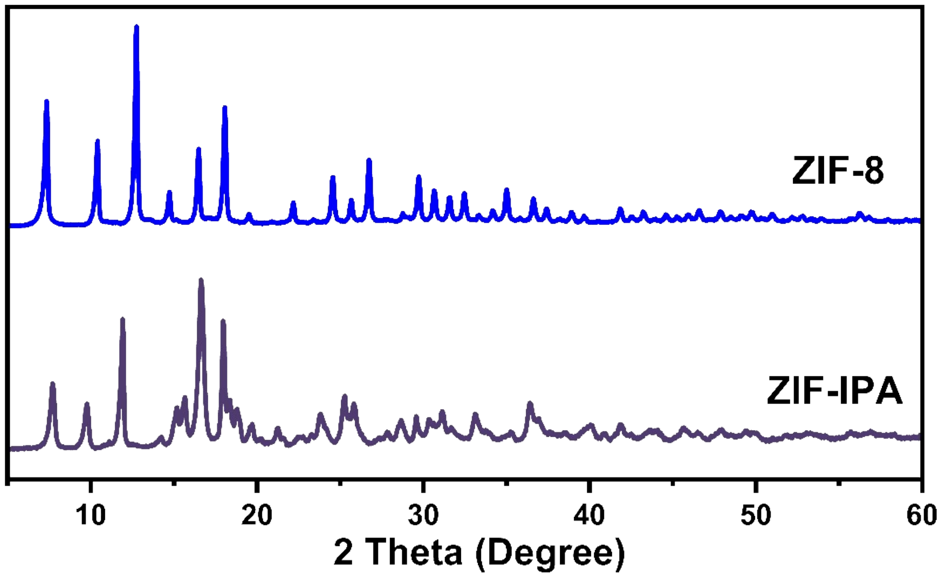

Figure S15. XRD comparison between ZIF-8 and ZIF-IPA (synthesized following the same protocol that for Mel-ZIF and Tap-ZIF but in the absence of heterocyclic amine).

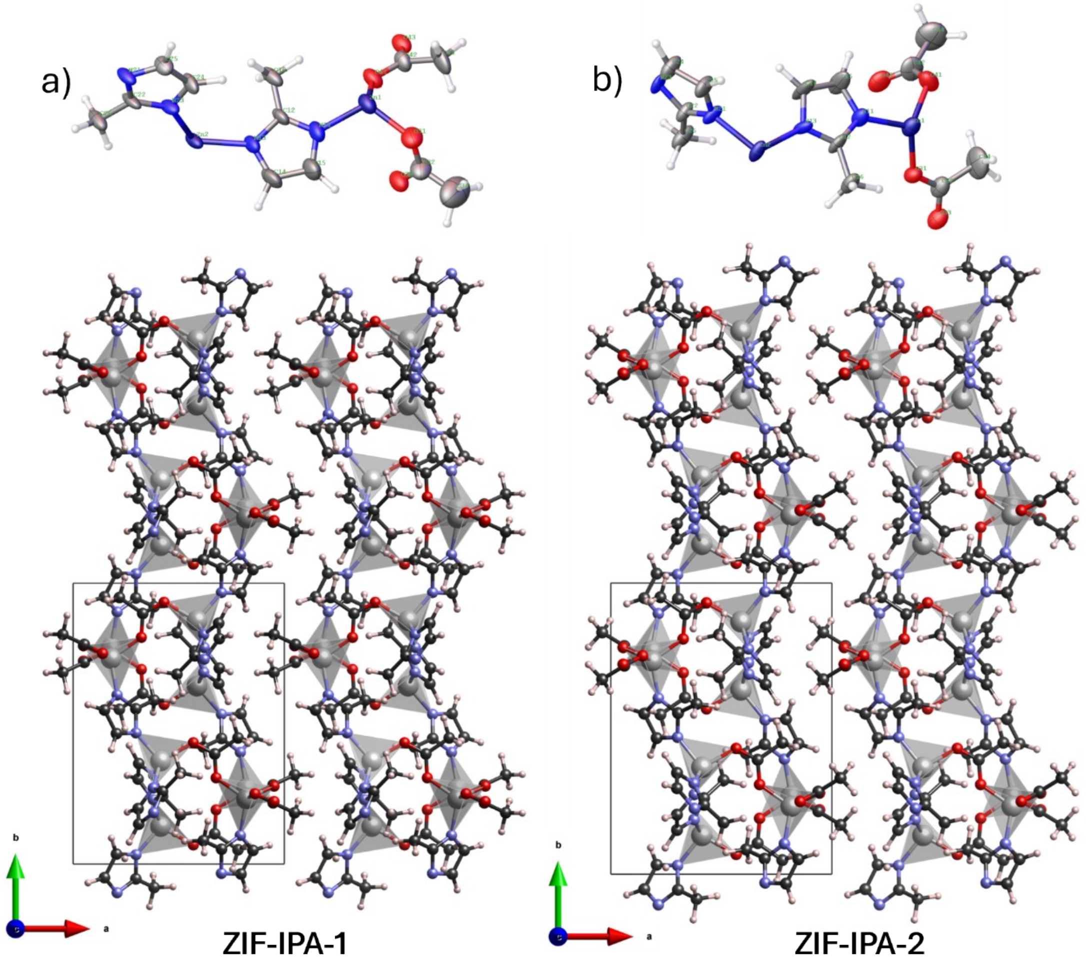

Figure S16. Asymmetric unit of ZIF-IPA (top) and crystal structure of ZIF-IPA viewed along the *c* axis for a) ZIF-IPA-1 and b) ZIF-IPA-2, showing the stacking of the layers along the *a* axis.

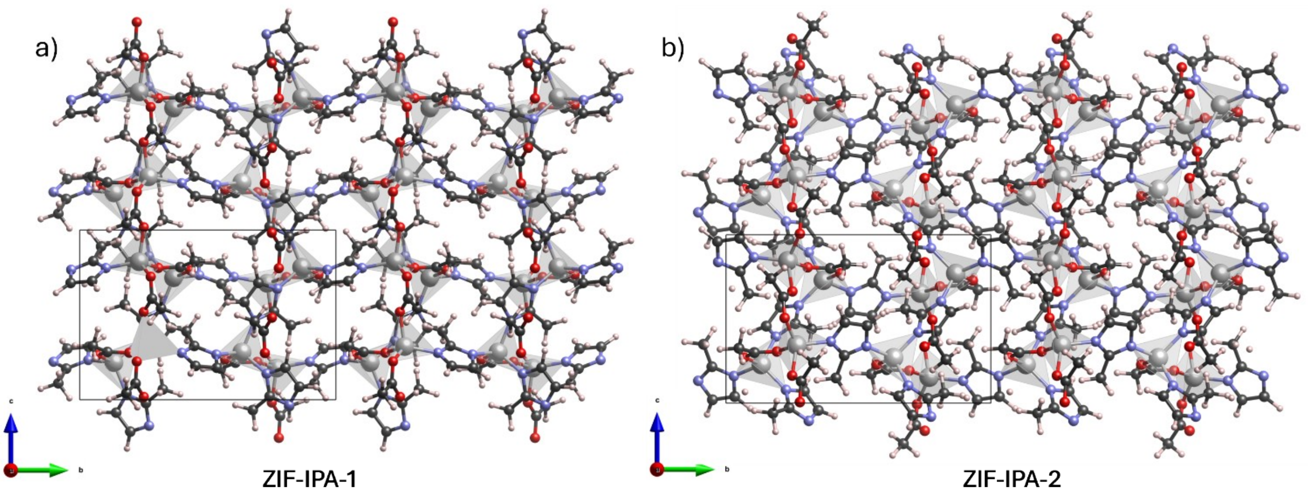

Figure S17. Crystal structure of ZIF-IPA viewed along the *a* axis for a) ZIF-IPA-1 and b) ZIF-IPA-2, showing the '*open-pore'* and '*closed-pore'* conformations due to the different orientations of the imidazole and acetate ligands.

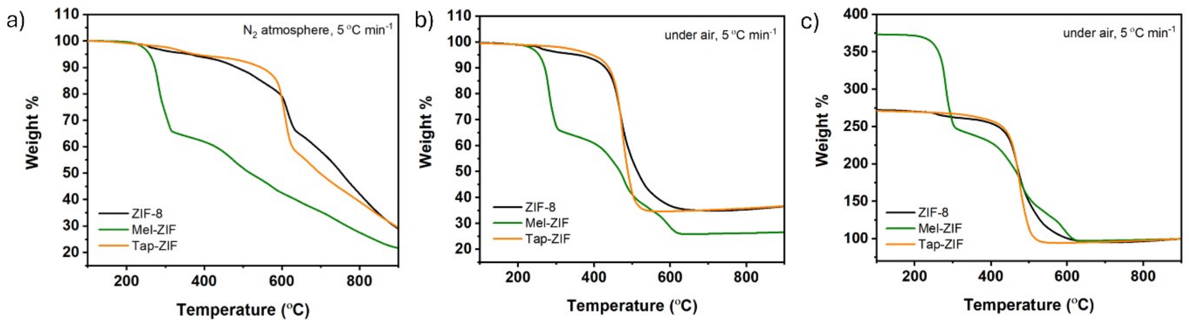

Figure S18. Thermogravimetric analysis in N_2 and O_2 of ZIF-8, Mel-ZIF and Tap-ZIF.

Table S5. Comparison of carbonized ZIF, Tap-ZIF and Mel-ZIF at 1000 °C in terms of BET specific surface area, micropore volume and mesopore volume.

Sample	BET area (m^2 g^{-1})	Micropore volume (cm^3 g^{-1})	Mesopore volume (cm^3 g^{-1})
**ZIF-81000**	691 ± 1	0.260	0.090
**Tap-ZIF1000**	858 ± 2	0.307	0.116
**Mel-ZIF1000**	828 ± 2	0.306	0.115

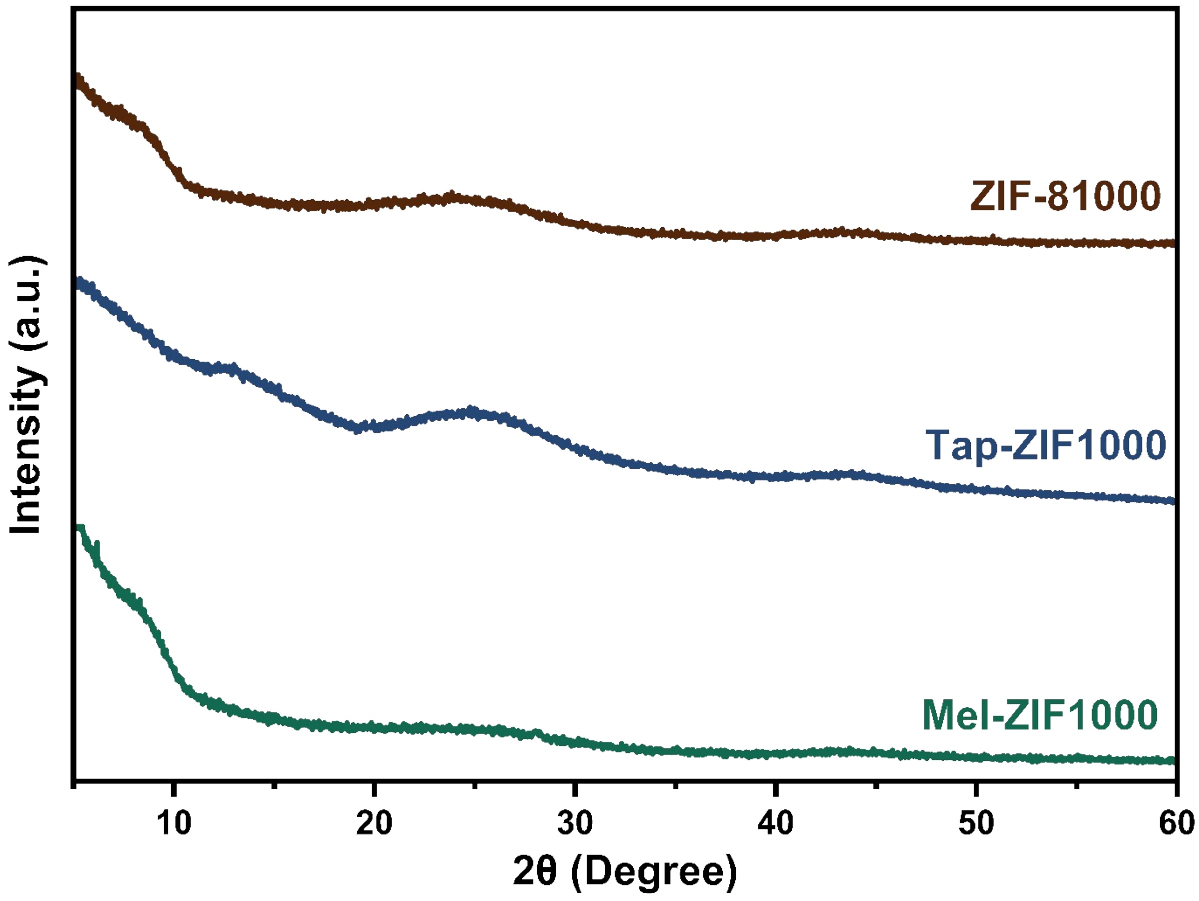

Figure S19. XRD patterns of pyrolyzed materials.

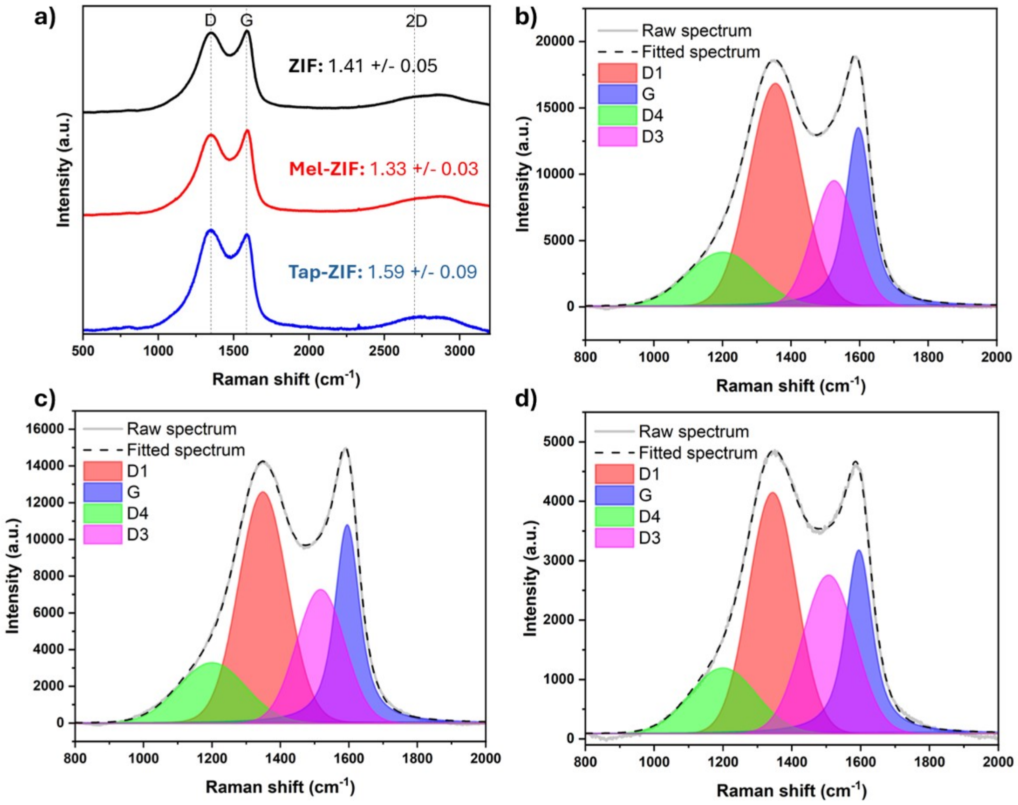

Figure S20. a) Averaged Raman spectra for the carbonised ZIF-derived materials with mean I_D/I_G values and standard deviation. Examples of spectrum deconvolution for carbonised b) ZIF-8, c) Mel-ZIF and d) Tap-ZIF.

Table S6. Chemical composition in atomic and weight percent of ZIF-derived materials obtained via XPS.

Chemical Composition								
	C1s		N1s		O1s		Zn2p	
	at %	wt %	at %	wt %	at %	wt %	at %	wt %
ZIF-81000	74.2	62.7	18.6	18.3	4.1	4.6	3.1	14.4
Tap-ZIF1000	68.1	53.8	22.7	20.9	4.4	4.7	4.8	20.7
Mel-ZIF1000	73.5	61.3	19.3	18.8	4.4	5.1	2.8	14.8

Table S7. Relative abundance and binding energies of N 1*s* XPS peaks.

Relative abundance of each N1s peak %					
	Pyridinic (BE 398.34 eV)	Pyrrolic (BE 400.77 eV)	Zn-N (BE 399.49 eV)	Graphitic (BE 402.69 eV)	N-O (BE 404.98 eV)
ZIF-8 1000	27.0	34.4	25.0	9.9	3.7
Tap-ZIF1000	51.5	27.2	6.1	9.6	5.6
Mel-Zif 1000	44.8	34.6	7.4	9.4	3.8

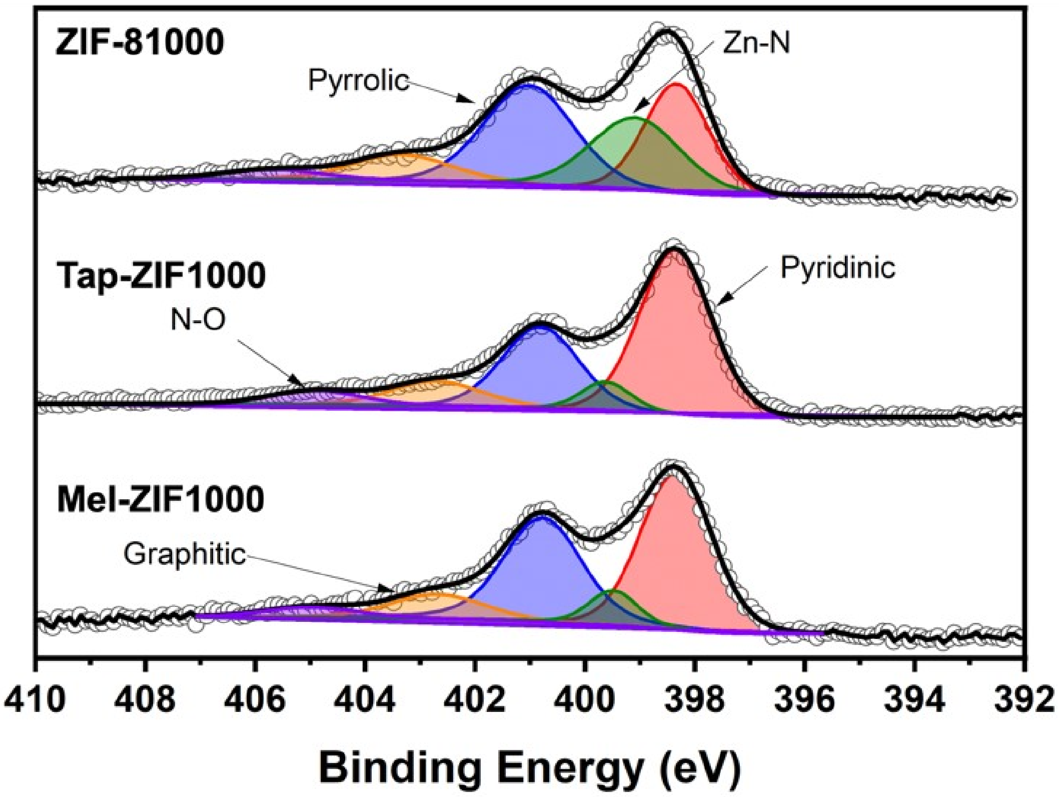

Figure S21. N1s XPS spectra of ZIF-derived materials.

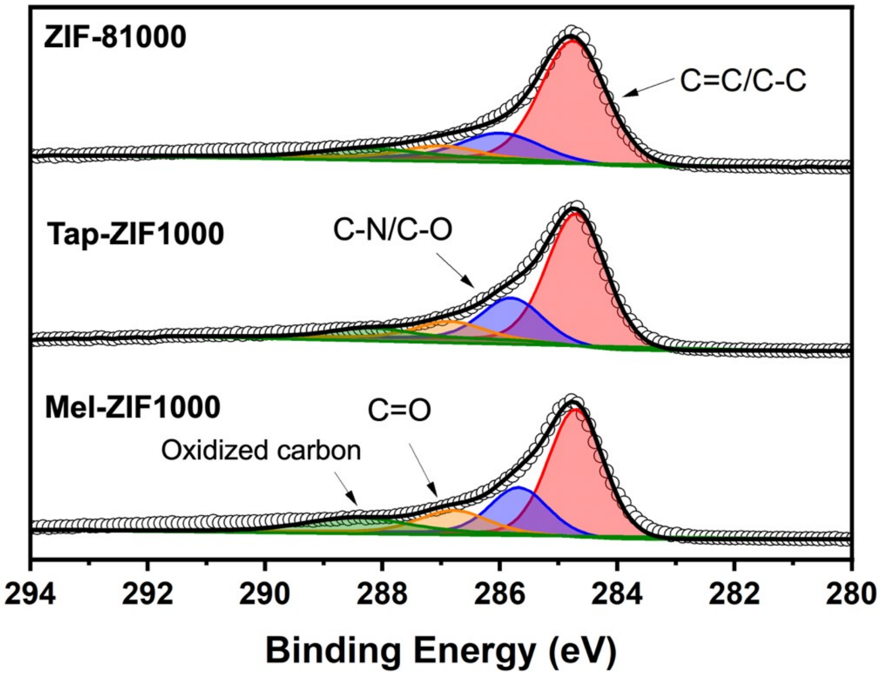

Figure S22. C1s XPS spectra of ZIF-derived materials.

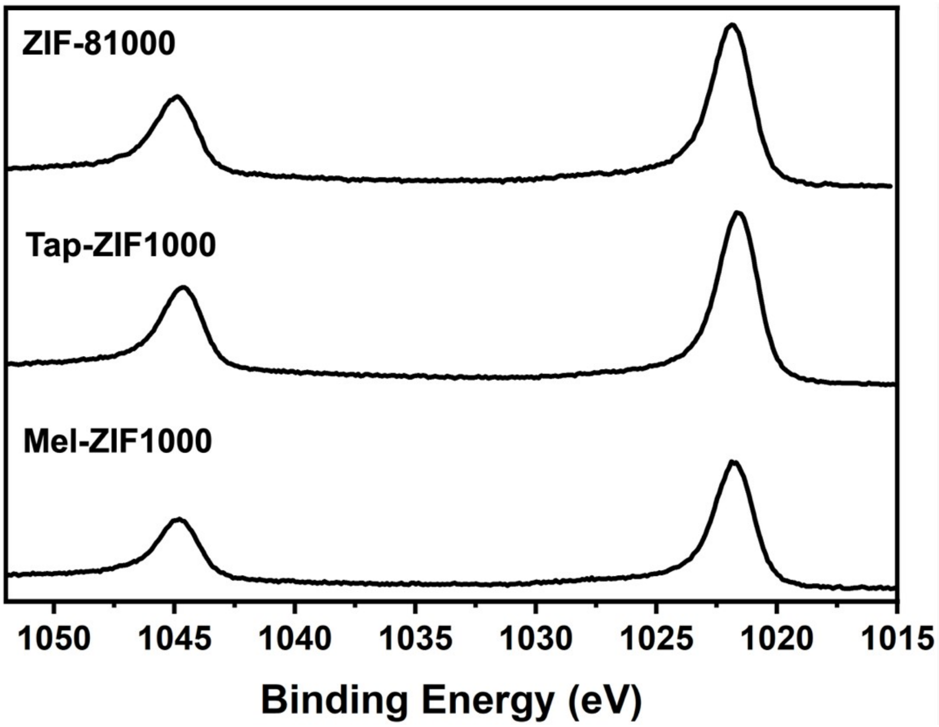

Figure S23. Zn 2*p* XPS spectra of ZIF-derived materials.
